# CARM1/PRMT4 facilitates XPF–ERCC1 heterodimer assembly and maintains nucleotide excision repair activity

**DOI:** 10.1093/nar/gkaf355

**Published:** 2025-04-30

**Authors:** Hiroyuki Niida, Masahiko Ito, Kenta Iijima, Akira Motegi, Rin Ogihara, Hironobu Akiyama, Chiharu Uchida, Satoshi Sakai, Tatsuya Ohhata, Atsushi Hatano, Michiko Hirose, Atsuo Ogura, Masaki Matsumoto, Neil Q McDonald, Masatoshi Kitagawa

**Affiliations:** Department of Molecular Biology, Hamamatsu University School of Medicine, 1-20-1 Handayama, Hamamatsu, Shizuoka 431-3192, Japan; Department of Microbiology and Immunology, Hamamatsu University School of Medicine, Hamamatsu, Shizuoka 431-3192, Japan; Laboratory Animal Facilities and Services, Institute of Photonics Medicine, Hamamatsu University School of Medicine, 1-20-1 Handayama, Hamamatsu, Shizuoka 431-3192, Japan; Department of Radiation Genetics, Kyoto University Graduate School of Medicine, Yoshida-Konoe-cho, Kyoto 606-8501, Japan; Department of Molecular Biology, Hamamatsu University School of Medicine, 1-20-1 Handayama, Hamamatsu, Shizuoka 431-3192, Japan; Department of Molecular Biology, Hamamatsu University School of Medicine, 1-20-1 Handayama, Hamamatsu, Shizuoka 431-3192, Japan; Advanced Research Facilities and Services, Institute of Photonics Medicine, Hamamatsu University School of Medicine, 1-20-1 Handayama, Hamamatsu, Shizuoka 431-3192, Japan; Department of Molecular Biology, Hamamatsu University School of Medicine, 1-20-1 Handayama, Hamamatsu, Shizuoka 431-3192, Japan; Department of Molecular Biology, Hamamatsu University School of Medicine, 1-20-1 Handayama, Hamamatsu, Shizuoka 431-3192, Japan; Department of Omics and Systems Biology, Graduate School of Medical and Dental Sciences, Niigata University, Niigata 951-8510, Japan; Bioresource Engineering Division, RIKEN BioResource Research Center, Ibaraki 305-0074, Japan; Bioresource Engineering Division, RIKEN BioResource Research Center, Ibaraki 305-0074, Japan; Department of Omics and Systems Biology, Graduate School of Medical and Dental Sciences, Niigata University, Niigata 951-8510, Japan; Institute of Structural and Molecular Biology, School of Natural Sciences, Birkbeck College, Malet Street, London WC1E 7HX, United Kingdom; Signalling and Structural Biology Laboratory, Francis Crick Institute, London NW1 1AT, United Kingdom; Department of Molecular Biology, Hamamatsu University School of Medicine, 1-20-1 Handayama, Hamamatsu, Shizuoka 431-3192, Japan; Molecular Targeting Laboratory, Institute of Photonics Medicine, Hamamatsu University School of Medicine, 1-20-1 Handayama, Hamamatsu, Shizuoka 431-3192, Japan

## Abstract

The structure-specific endonuclease, XPF–ERCC1, plays a central role in DNA damage repair. This nuclease is known to be important for nucleotide excision repair, interstrand crosslink repair, and DNA double-strand repair. We found that the arginine methyltransferase, CARM1/PRMT4, is essential for XPF stabilization and maintenance of intracellular protein levels. Loss of CARM1 results in a decrease in XPF protein levels and a concomitant decrease in ERCC1 protein. A similar destabilization of XPF protein was observed in cells expressing a mutant in which XPF arginine 568 was replaced by lysine. Loss of CARM1 impaired XPF–ERCC1 accumulation at the site of damage and delayed removal of cyclobutane pyrimidine dimers by UV. As a result, CARM1-deficient cells showed increased UV sensitivity. Our results provide insight into the importance of CARM1 not only in the mechanism of XPF–ERCC1 complex stabilization but also in the maintenance of genome stability.

## Graphical Abstract

**Graphical Abstract:**
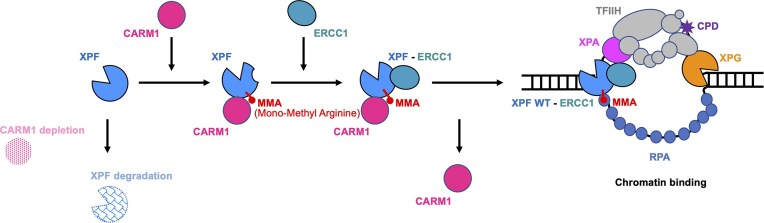

## Introduction

DNA damage in human cells is caused by a variety of factors, including intracellular reactive oxygen species (ROS), DNA replication, and external stimuli such as ultraviolet light (UV) and ionizing radiation. When DNA damage is repaired by DNA repair mechanisms, homeostasis is maintained, but when DNA damage cannot be repaired, cells remove DNA-damaged cells from tissues through apoptosis. However, mutations or chromosomal deletions during DNA repair can lead to cancerous transformation of cells.

UV radiation damage mainly generates pyrimidine dimers, which distort the higher-order structure of DNA, thus inhibiting DNA replication and transcription. This type of DNA damage is repaired by nucleotide excision repair (NER). In addition to UV-induced DNA damages, NER removes a cisplatin-bound DNA (DNA adducts) [1, 2]. In this repair mechanism, the damaged DNA is detected as a distortion of the higher-order structure of DNA, and the single-stranded DNA containing the causative damage is removed. There are two different pathways used for damage recognition. One pathway, called transcription-coupled NER, operates when DNA damage occurs on the template DNA that is being transcribed [3]. In this pathway, the DNA damage triggers inhibition of the progression of RNA polymerase II during transcription to transmit repair signals via repair factors such as Cockayne syndrome type A, Cockayne syndrome type B, and UV-stimulated scaffold protein A. Another pathway is the global-genome NER (GG-NER). This pathway is initiated by the recognition of DNA damage by xeroderma pigmentosum group C protein (XPC) [4]. XPC alone cannot recognize relatively small DNA damage such as cyclobutane pyrimidine dimers (CPDs) [5–7]. Hence, damage-specific DNA binding protein 2 (DDB2), which is a substrate recognition subunit of DDB1–CUL4–RBX1 (CRL4) ubiquitin ligase, recognizes CPDs and facilitates recruitment XPC to the damage sites. Once XPC is loaded onto the damaged site, the repair signal is transmitted downstream, and transcription factor II H (TFIIH), a basic transcription factor consisting of 10 subunits such as xeroderma pigmentosum group B protein, xeroderma pigmentosum group D protein, and xeroderma pigmentosum group A protein (XPA), is loaded onto the damaged site. XPD scans DNA from 5′ to 3′ [8], and XPA confirms the damage [9], and then the single-stranded DNA harboring the damaged site is cleaved by xeroderma pigmentosum group F protein (XPF) and xeroderma pigmentosum group G protein (XPG) [10]. The resulting gap is filled with DNA polymerase to complete the repair.

XPF–excision repair cross-complementation group 1 (ERCC1) is a well-conserved endonuclease that nicks substrate with specific structure at the 5′ end of junctions between single-stranded and double-stranded DNA. XPF and ERCC1 are thought to be paralogs resulting from gene duplication and bind to each other through both their central nuclease/nuclease-like domain and a helix–hairpin–helix (HhH_2_) domain at their C-termini [11–14]. This binding is necessary for the stability of both proteins. In addition to the central nuclease domain and HhH_2_ domain, the XPF protein contains an N-terminal helicase-like module (HLM) [15]. HLM binds replication protein A (RPA) to localize XPF–ERCC1 the appropriate site of damage during DNA repair [16]. On the other hand, binding of ERCC1 to XPA is essential for XPF–ERCC1 recruitment to the site of damage [17].

Mutations in XPF–ERCC1 have been associated with several diseases, including Cockayne syndrome, xeroderma pigmentosum, cerebro-oculo-facio-skeletal syndrome, and Fanconi anemia [18–22]. Many of the XPF–ERCC1 mutations found to date disrupt heterodimer formation and cause intracellular mislocalization [23].

Arginine methylation, like phosphorylation and ubiquitination, is an abundant post-translational modification and also serves as an epigenetic marker. There are three types of arginine methylation, monomethyarginine (MMA), asymmetric dimethylarginine (ADMA), and symmetric dimethylarginine mediated by protein arginine methyltransferases (PRMTs) [24]. PRMT regulates various cellular processes, including transcription, signal transduction, and DNA damage responses, by methylating histone and nonhistone substrates [24, 25]. For example, coactivator-associated arginine methyltransferase 1 (CARM1), also known as PRMT4, is a histone methyltransferase and is well known to act as a transcriptional co-activator for transcriptional activation of p53 response genes [26]. PRMT1 methylates glycine–arginine-rich domain of meiotic recombination 11 (MRE11) homolog A to regulate its exonuclease activity [27]. CARM1/PRMT4, together with PRMT2, regulates transcription and double-strand break repair by performing arginine methylation of bromodomain containing 4, which functions as an epigenetic reader and plays an important role in the regulation of transcription and genome stability, regulating its binding to acetylated histones [28]. On the other hand, there are no reports that CARM1 maintains the cellular DNA repair function by methylating DNA repair factors as substrates.

In this study, we screened for essential proteins required for GG-NER in cells using a small interfering RNA (siRNA) library and found that CARM1 is involved in the elimination of CPD after UV irradiation. We found that CARM1 monomethylates multiple arginines in XPF. Inhibition of this methylation by CARM1 depletion or by mutants in which arginine was replaced by lysine suppressed heterodimer formation between XPF and ERCC1. These results indicate that methylation of XPF by CARM1 promotes stable complex formation with ERCC1 and allows this endonuclease to function in a critical step of GG-NER.

## Materials and methods

### Cell culture

HeLa (JCRB Cell Bank) was established from cancer cells of the uterine cervix, HEK293 (JCRB Cell Bank) was established from kidney cells, and XP2YO-SV (JCRB Cell Bank) was established from a hereditary disease with a high cancer risk, xeroderma pigmentosum (autosomal recessive), complementation group F, an SV40-transformed skin fibroblast. All cells were cultured in Dulbecco’s modified Eagle’s medium (DMEM) supplemented with 10% fetal bovine serum (FBS) and penicillin/streptomycin at 37°C with 5% CO_2_. METTL23 Knockout Mouse Embryonic Fibroblasts (KO MEFs) were established from the 13.5 embryos of METTL23 homozygous mice [29]. Immortalized METTL23 KO MEFs were established by subculture for 3 months. All cells tested negative for mycoplasma.

**Table 1.** Antibody

Antibody	Maker	Catalog no.	Reference
FLAG	Merck, Darmstadt, Germany	F1804	
CARM1	Cell Signaling Technology, Danvers, MA, USA	12495	
XPF	Cell Signaling Technology, Danvers, MA, USA	13465	
XPC	Santa Cruz Biotechnology, Dallas, TX, USA	sc-74410	
phospho Ser50 and Ser53 HBO1			See ref. [38]
Myc (9E10)	Santa Cruz Biotechnology, Dallas, TX, USA	sc-40	
ERCC1 (D-10)	Santa Cruz Biotechnology, Dallas, TX, USA	sc-17809	
ORC2	Santa Cruz Biotechnology, Dallas, TX, USA	sc-13238	
a-Tubulin	Santa Cruz Biotechnology, Dallas, TX, USA	sc-8035	
Mono-methyl arginine [mme-R]	Cell Signaling Technology, Danvers, MA, USA	8015	
Asymmetric di-methyl arginine motif [adme-R]	Cell Signaling Technology, Danvers, MA, USA	13522	
GST	Santa Cruz Biotechnology, Dallas, TX, USA	sc-138	
CPD	Cosmo Bio, Tokyo, Japan	NMDND001	
TFIIH p89	Santa Cruz Biotechnology, Dallas, TX, USA	sc-293	
XPG	Santa Cruz Biotechnology, Dallas, TX, USA	sc-189	
β-actin	Santa Cruz Biotechnology, Dallas, TX, USA	sc-69879	

### Antibodies

A list of antibodies used in this study is provided in Table 1.

### siRNA

A list of siRNAs used in this study is included in Table 2.

**Table 2.** siRNA and oligo DNA

siRNA	Sequence	Reference
siCARM1	GCUUUCAUCGGCUCCAUAAtt	
*Primer for making GST substrates*		
GST-R180-S	CCGAATTCCGCTTTCACAGACAATGCTGTT	
GST-R180-AS	GCCGCTCGAGCACAAAAAGATTTCTCATCACTC	
GST-R267-S	CCGAATTCCTCTTTAGAAAATGCTATTGGAAAACC	
GST-R267-AS	GCCGCTCGAGTCCAAGCTGGTGCCACAAAG	
GST-R415-S	CCGAATTCCAATAAGGAGAGTGAAGCT	
GST-R415-AS	GCCGCTCGAGCTGGGAACATGTTCGGTC	
GST-R421-S	CCGAATTCCACATGTTCCCAGCTGAG	
GST-R421-AS	GCCGCTCGAGCAATAAGAAGGCCTCCG	
GST-R434-S	CCGAATTCCGACTATATCACTCTTGGAGC	
GST-R434-AS	GCCGCTCGAGTTCAGCTTTGCTATCCTTCT	
GST-R568-S	CCGAATTCCCTCACTATCATCCATCCGCT	
GST-R568-AS	GCCGCTCGAGTCTTGGCTCCACTTCATGTA	
GST-R670-S	CCGAATTCCGACCTAGTAAGAGGCACAGCA	
GST-R670-AS	GCCGCTCGAGTGTACCATTCTGTTCCTGGC	
GST-R726-S	CCGAATTCCGAGGTTGGAGATTACATCCT	
GST-R726-AS	GCCGCTCGAGATTTAAAGAGCCGATTAAATCAC	
GST-R750-S	CCGAATTCCAACGGCCGCCTCTACAGC	
GST-R750-AS	GCCGCTCGAGAGGGTCAAACTCAATCAGAA	
GST-RK less-S	CCGAATTCCCTCCTCTACCACTTTCTCCA	
GST-RK less-AS	GCCGCTCGAGCTCCTCGGCCGGCTGCGT	
*Primer for making KO cell*		
ERCC4-CRISPR-5-S	CACCGCTATATGCCCAGTCTAGAA	
ERCC4-CRISPR-5-AS	AAACTTCTAGACTGGGCATATAGC	
ERCC4-CRISPR-3-S	CACCGATGTCACTGGCAATAATGCG	
ERCC4-CRSPR-3-AS	AAACCGCATTATTGCCAGTGACATC	
CARM1-CRISPR-5-S	CACCGCCTCCATGCTAGAACTGCGG	
CARM1-CRISPR-5-AS	AAACCCGCAGTTCTAGCATGGAGGC	
CARM1-CRISPR-3-S	CACCGTCTCTCATGGCCGGTAGTCA	
CARM1-CRISPR-3-AS	AAACTGACTACCGGCCATGAGAGAC	
*Primer for RT-qPCR*		
xpf qpcr-F	CACCTCTTTTGCAGAAGTC	
xpf qpcr-R	GATGACCGGTATGCATATT	
GAPDH-F	GATTCCACCCATGGCAAATT	
GAPDH-R	CTTCCCGTTCTCAGCCTTGA	
*Primer for nuclease assay*		
Fluorescent control	ATAGTCGGTTATGTATCTAGCGAGATAAAGTGTAGATGCAGCGTGGACAT	
Unlabeled control	TACAGGTGCGACGTAGATGTGTTTATCTCGCTAGATACATAACCGACTAT	

### Oligo DNA

A list of oligo DNA used in this study is included in Table 2.

### Plasmids

CARM1-FLAG-WT expression plasmid (FLAG/pcDNA3) was described in a previous report [29]. CARM1-FLAG-AAA expression plasmid was mutated at amino acid 189–191 LDV to AAA to make an inactive mutant referred to hereafter as CARM1 AAA [30]. XPF-Myc expression plasmid (pEF6/Myc-His vector) was described in a previous report [31]. Glutathione S-transferase (GST)-XPF fragment plasmids were made by inserting the DNA from the region containing the arginine to be methylated into pGEX 4T-3 (Addgene, Watertown, MA, USA). To make retrovirus expressing XPF-WT, 6RK, R180K, R568K, and R726KR750K, each XPF complementary DNAs were inserted into pRetroX-TetOne-Puro plasmid (Takara Bio Inc., Shiga, Japan). For the expression of recombinant XPF proteins using the wheat germ expression (WGE) cell-free expression system (CellFree Sciences, Kanagawa, Japan), the XPF-Myc-His fragment was cloned into the pEU-E01-MCS vector (CellFree Sciences) from the pEF6/XPF-Myc-His vector.

### DOX-induced XPF expression cells

Doxycycline (DOX)-induced XPF expression cells were established according to the manufacturer’s protocol (Takara Bio Inc., Shiga, Japan).

### Knockout HeLa cells

Annealed sense and antisense oligo DNAs were subcloned into pSpCas9(BB)-2A-Puro (PX459 Puro) plasmid (Addgene, Watertown, MA, USA). CARM1-5′ and CARM1-3′ plasmids or XPF-5′ and XPF-3′ plasmids were transfected into HeLa cells. After 24 h transfection, cells were selected with 1 μg/ml puromycin for 48 h. After puromycin selection, cells were cultured in normal medium for 3 days, and then 24 colonies per each transfection were picked up in 96-well plates. Cells were expanded and lack of expression of the target protein was confirmed by western blotting.

### siRNA library screening

Five thousand HeLa cells were incubated overnight in a 96-well plate. Then, 1 pmol of the Radiation Biology Center siRNA library (Ambion, Austin, TX, USA) was transfected using Lipofectamine 2000 (Invitrogen, Carlsbad, CA, USA) and incubated for 48 h. After 24 h of incubation following 15 J m^−2^ UV irradiation, the cells were fixed with 4% paraformaldehyde at room temperature for 10 min and washed twice with phosphate-buffered saline (PBS). Next, the cells were treated with 0.15% Triton X-100 for 5 min and washed twice with PBS. The cells were rinsed once with distilled water and denatured with 2 M HCl at room temperature for 30 min. The cells were washed five times with PBS and blocked with blocking buffer (20% FBS/0.1% Triton X-100 in PBS) for 1 h. The cells were incubated with CPD antibody (1:2000 dilution with 3% FBS in PBS) for 60 min at room temperature and then washed once with PBS. The cells were stained with secondary antibodies conjugated with Alexa Fluor 488 fluorescent dyes (1:500 dilution with 3% FBS in PBS) for 30 min. After washing twice with PBS, the fluorescence intensity was quantified using an IN cell analyzer 2200 (GE Healthcare Life Sciences, Marlborough, MA, USA). Relative intensities of CPDs were normalized to DAPI.

### CPD repair assay

CARM1 KO HeLa cells were transfected with CARM1 WT-FLAG or AAA-FLAG for 48 h. XP2YO-SV cells were treated with 1 μg/ml DOX for 24 h. Then, the parental, XPF KO, CARM1 KO, CARM1 KO transfected CARM1 WT-FLAG and AAA-FLAG cells, and XP2YO-SV cells were irradiated with 12 and 4 J m^−2^ UV, respectively. HeLa and XP2YO-SV cells were cultured for 24 and 8 h, respectively, fixed, and immunostained with an anti-CPD antibody. DNA was counterstained with 4,6-diamidino-2-phenylindole (DAPI). The relative intensity of CPD was normalized to that of DAPI. Relative remaining CPD was expressed as a percentage of 0 h condition.

### LC–MS/MS analysis

XPF-Myc, ERCC1, and CARM1-FLAG expression vectors were transfected into HEK293 cells and incubated for 48 h. The harvested cells were suspended in His-Lysis buffer (20 mM Tris–HCl, 0.5 M NaCl, 0.5% NP-40 with protease inhibitor) and sonicated for 60/60 s on/off. After centrifugation at 12 000 rpm for 10 min, the supernatant was mixed with 50 μl anti-Myc tag mAb magnetic beads (MBL, Japan). The cells were immunoprecipitated overnight and washed six times with His-Lysis buffer without NP-40. The immunoprecipitates were suspended in 30 μl 2× sample buffer and boiled for 3 min. Myc IP samples were separated by 7.5% SDS–PAGE and stained according to the protocol of the silver stain MS kit (Fujifilm, Japan). The 104 kDa XPF-Myc band was excised from the gel and digested with trypsin. Trypsin digestion was performed as follows: the gels were suspended in 150 μl of acetone and dried in a speed-vac. The gels were incubated in 100 μl of 100 mM ammonium bicarbonate (AB)/10 mM dithiothreitol (DTT) at 56°C for 45 min, the supernatant was removed, 100 μl of 100 mM AB/55 mM iodoacetamide was added, and the gels were incubated at room temperature in the dark for 30 min. The supernatant was removed, and the gels were washed twice with 100 mM AB, after which 100 μl of 100 mM AB/50% acetone was added and the gels were dried in a speed-vac for 1 h. Next, 2 μl of 12.5 ng/μl trypsin was added, incubated on ice for 30 min, and then 20 μl of 25 mM AB was added and incubated at 37°C overnight. One hundred microliters of 0.1% trifluoroacetic acid (TFA) was added, the mixture was vortexed, and then sonicated for 30/30 s on/off. The supernatant was collected. Next, 100 μl of 0.1% TFA/30% acetone was added, the mixture was vortexed, and then sonicated. The supernatant was collected. Next, 100 μl of 0.1% TFA/60% acetone was added, the mixture was vortexed, and then sonicated. The supernatant was collected. The supernatant was combined, and the sample dried in a speed-vac was analyzed by LC–MS/MS.

Samples were analyzed using Q Exactive or Q Exactive plus mass spectrometer (Thermo Fisher Scientific, USA) equipped with a Dionex Ultimate 3000 high-performance liquid chromatography system (Dionex Corporation) via a nano-electrospray source with a column oven set at 42°C (AMR Inc., Japan). Peptides were injected to a pre-column (L-column micro, Chemicals Evaluation and Research Institute) and separated by an in-house made 20 cm column packed with 2 μm octadecyl silane particle (Chemicals Evaluation and Research Institute, Japan). The separation was typically performed with a linear gradient of 5%–45% solvent B over 50 min, 45%–90% B over 1 min, and held at 90% B for 10 min at a flow rate of 200 nl/min, where solvent A is 0.1% formic acid and solvent B is 0.1% formic acid in acetonitrile. Data acquisition was conducted in data-dependent acquisition mode. MS spectra were acquired with scan ranges set at *m*/*z* 375–1600 or 350–1600, and MS/MS spectra were acquired with a scan range of *m*/*z* 200–2000. MS spectra were acquired at a resolution of 70 000 at *m*/*z* 400 after accumulation to a target value of 1 × 10^6^ with the maximum ion injection times for 60 ms. Up to the top 10 most abundant ions with charge of 2^+^, 3^+^, or 4^+^ from the survey scan were selected with an isolation window of 1.5 *m*/*z* and fragmented with an automatically optimized collision energy. MS/MS spectra were acquired at a resolution of 17 500 at *m*/*z* 400 after accumulation to a target value of 5 × 10^4^ with the maximum ion injection times for 120 ms.

Raw data obtained from MS analyses were processed using Proteome Discoverer software 3.1 (Thermo Fisher Scientific, USA). The search parameters were set as follows: Trypsin/P was selected as the enzyme, allowing cleavage at the carboxyl side of lysine and arginine, even when followed by proline. The allowed number of missed cleavages was set to 2, and carbamidomethylation of cysteine was selected as fixed modification. Variable modifications were set to mono- and dimethylation of arginine residues, oxidation of methionine residues, acetylation of the protein N-terminus, and N-terminal methionine excision. The maximum number of missed cleavages was set to 2.

### Subcellular fractionation

Subcellular fractionation of HeLa or XP2YO-SV cells was performed as follows. Briefly, 6 × 10^6^ cells were suspended in 200 μl of solution A (10 mM HEPES, pH 7.9, 10 mM KCl, 1.5 mM MgCl_2_, 0.34 M sucrose, 10% glycerol, 1 mM DTT, and protease and phosphatase inhibitors) with Triton X-100 at a final concentration of 0.1%. The cells were incubated on ice for 5 min and then centrifuged at 1300 × *g* for 4 min to collect the cytoplasmic fractions. The isolated nuclei were then washed with solution A, lysed with 200 μl of solution B (3 mM ethylenediaminetetraacetic acid (EDTA), 0.2 mM ethylene glycol tetraacetic acid (EGTA), 1 mM DTT, and protease and phosphatase inhibitors), and incubated on ice for 10 min. The soluble nuclear fractions were collected by centrifugation at 1700 × *g* for 4 min. Then cytoplasmic and nuclear fractions were mixed as a soluble fraction. The isolated chromatin was washed with solution B, centrifuged at 10 000 × *g* for 1 min, and then resuspended in 200 μl sample buffer. Then, the chromatin fraction was sonicated four times for 30 s each using a Bioruptor (Sonicbio, Samukawa, Kanagawa, Japan).

### Western blotting

Cells were lysed with lysis buffer. Cell lysates were centrifuged at 12 000 × *g* for 10 min at 4°C, and the protein-containing supernatant was collected. The samples were loaded onto an SDS–PAGE gel. After electrophoresis, proteins separated in the gel were transferred onto a PVDF membrane. The membranes were blocked at room temperature for 1 h using 5% dry skim milk in Phosphate Buffered Saline with 0.3% Tween 20 (PBST), followed by incubation with the primary antibody at 4°C overnight. After washing three times with PBST, a horseradish peroxidase-conjugated secondary antibody was applied at room temperature for 60 min. A chemiluminescent substrate (Bio-Rad, USA) was added to the membrane in accordance with the manufacturer’s instructions. The signals were captured using a CCD camera-based imager (Vilber Bio Imaging, Singapore).

### Co-immunoprecipitation

Approximately 2 × 10^7^ HEK293 cells were lysed with 450 μl 20 mM NaCl buffer (25 mM Tris–HCl, pH 7.5, 0.5% NP-40, 20 mM NaCl, 2 mM MgCl_2_, and protease and phosphatase inhibitors) and sonicated five times for 5 s each using a Bioruptor. Cell lysates were centrifuged at 13 000 rpm for 15 min. Supernatants were collected, and 200 μl lysates were incubated with 5 μl anti-Myc or FLAG antibody with normal rabbit IgG. Immunoprecipitates were collected with protein G agarose and subjected to western blotting.

### Reverse transcription quantitative polymerase chain reaction analysis

Total RNA was isolated from cells using ISOGEN (NIPPONGENE, Japan), followed by reverse transcription using oligo dT primer and reverse transcriptase SuperScript II (Invitrogen, USA). Quantitative PCR was carried out on a StepOnePlus system (Life Technologies, Carlsbad, CA, USA) using SYBR^®^ Green Realtime PCR Master Mix (TOYOBO, Osaka, Japan). Each expression value was normalized with GAPDH. Primer sequences are listed in Table 2.

### Global and local UV irradiation

For global UV irradiation, UV-C light irradiation was applied using a UV lamp (FUNA UV Cross-linker FS-800; 254 nm UV, Funakoshi, Tokyo, Japan) after washing with PBS. For local UV irradiation, cells were washed once with PBS and covered with a polycarbonate isopore membrane filter (8 μm pore size). Subsequently, UV-C light irradiation was performed using five low-pressure mercury lamps (Toshiba, Tokyo, Japan) at a dose rate of 0.43 J m^−2^s^−1^ for 120 s (∼50 J m^−2^). Immediately after UV irradiation, the cells were incubated in DMEM supplemented with 10% FBS for 30 min at 37°C and then fixed.

### Clonogenic survival assay

HeLa cells were transfected with the indicated siRNAs. After 48 h, 500 cells were seeded in 10-cm dishes. XP2YO-SV cells were induced XPF by adding DOX. After 24 h, 2000 cells were seeded in 10-cm dishes. At 24 h post-incubation, the cells were irradiated with the indicated doses of UV light. At 10 days after seeding, colonies were fixed with 1:1 MeOH/acetic acid and stained with crystal violet. Finally, the number of colonies was counted, and the viable fractions were normalized by the plating efficiency of each cell line.

### Immunofluorescence

Cells were fixed in a 1:1 solution of methanol and acetone for 10 min and then washed three times with PBS. The cells were blocked in PBS with 10% FBS for 30 min. The cells were then washed once with PBS and stained with a primary antibody (1:100 dilution in PBS) for 1 h. After washing with PBS, the cells were stained with a secondary antibody (1:500 dilution in PBS) conjugated with Alexa Fluor 488 or 594 fluorescent dyes for 30 min with 1 μg/ml DAPI. Finally, the cells were washed with PBS and mounted in VECTASHIELD mounting medium (Vector Laboratories, Burlingame, CA, USA).

### Quantification of UV-irradiated lesions

To calculate the accumulation rate of the indicated factors in the irradiated area, the mean intensity of the irradiated area was divided by the baseline intensity (the intensity of the non-irradiated area of the same nucleus) and expressed as a relative intensity increase from the baseline (%). The mean fluorescence intensities in UV-irradiated and non-irradiated areas were quantified using ImageJ (NIH, Bethesda, MD, USA).

### Recombinant protein

GST-ERCC1 was expressed in *Escherichia coli* BL21 strain (TAKARA, Japan). GST-ERCC1 was purified as follows. Five hundred milliliters of GST-ERCC1 pGEX 4T-3 transfected BL21 was induced with 0.1 mM IPTG at 37°C for 2 h and then harvested by centrifugation at 5000 rpm for 5 min. The cell pellet was then suspended in 5 ml PBS/5 mg/ml lysozyme and incubated at 37°C for 10 min. Seven milliliters of lysis buffer (PBS/0.1 M EDTA, 1% TritonX) was added, and the cells were lysed by sonication. The lysate was centrifuged at 15 000 rpm for 10 min at 4°C, and the supernatant was harvested. Two milliliters of glutathione beads (50% slurry) were added and rotated at 4°C for 1 h. The beads were washed twice with 10 ml lysis buffer, three times with 10 ml PBSE (PBS/0.1 M EDTA), and twice with 50 mM HEPES (pH 8.0) and then eluted with 1 ml HEPES (pH 8.0)/1.5 mg/ml glutathione.

XPF WT and R568K-Myc were expressed using a WGE system following manufacturer’s instructions. After translation, the reaction was diluted into 5× volume of Ni-binding Buffer (20 mM sodium phosphate buffer, pH 7.6, 250 mM NaCl, NP-40 0.5%, 0.1% Brij-35, and 10 mM imidazole) and bound on Ni-NTA beads (Qiagen) for 3 h at 4°C with gentle rotation. Ni beads washed with Ni-binding buffer five times, and eluted by Ni-binding buffer supplemented with 200 mM imidazole. The eluted fraction was diluted with an equal volume of His-Lysis buffer (20 mM Tris, pH 8.0, 250 mM NaCl, 0.5% NP-40), and 80 μl of anti-Myc tag mAb magnetic beads were added and rotated overnight at 4°C. The beads were washed three times with 1 ml of His-Lysis buffer, then once with 1 ml of 50 mM Tris (pH 8.0), and eluted with 50 μl of 1 mg/ml Myc peptide by incubation at room temperature for 15 min twice.

### GST pull-down assay

To confirm the binding of XPF and CARM1 *in vitro*, the following GST pull-down assay was performed. XPF WT-Myc was overexpressed together with ERCC1 in HEK293 cells. The lysate was mixed with GST-CARM1 expressed and purified in BL21 strain, incubated, and then pulled down using MagneGST™ Glutathione particles (Promega, Hollow Road, Madison, WI, USA). After washing three times with His-Lysis buffer, pulled-down XPF WT-Myc was detected using western blotting. GST was used as a negative control. The binding of XPF WT or R568K to ERCC1 was tested as follows. XPF WT and R568K-Myc were *in vitro* translated using a WGE system. The reaction mixture was mixed with GST-ERCC1 expressed and purified in BL21 cells and incubated for 3 h at 4°C. After pull-down using MagneGST™ Glutathione particles, the mixture was washed three times with His–lysis buffer, and XPF was detected by western blotting.

### *In vitro* methylation assay

CARM1-FLAG plasmid was transfected into HEK293 cells for 48 h, and then cells were lysed with IP-kinase buffer (50 mM HEPES, pH 7.9, 150 mM NaCl, 1 mM EDTA, 2.5 mM EGTA, 10% glycerol, 0.1% Tween 20 with protease and phosphatase inhibitors). The lysate was immunoprecipitated using FLAG affinity gel (Sigma–Aldrich, St. Louis, MO, USA) for 1.5 h at 4°C, washed three times with IP-kinase buffer and 50 mM Tris–HCl (pH 8.0) once. The CARM1-FLAG magnetic beads were mixed with *in vitro* translated and purified XPF WT and R568K-Myc and 1 mM *S*-adenosylmethionine in 50 mM Tris–HCl (pH 8.0)/10 mM DTT, and incubated for 3 h at 30°C. The reaction mixture was pulled down with GST-ERCC1 and detected using western blotting.

### *In vitro* nuclease assay

*In vitro* nuclease assays were performed according to a previously reported paper [32]. In brief, XPF was co-expressed with ERCC1 in HEK293 cells and affinity-purified using Anti-Myc tag mAb magnetic beads. After washing three times with His-Lysis buffer, the beads containing 300 fmol of XPF were subjected to nuclease reaction (5 mM HEPES, pH 8.0, 40 mM NaCl, 10% glycerol, 0.5 mM dithiothreitol, 0.1 mg/ml bovine serum albumin, and 1 mM MnCl_2_,5 pmol DNA substrate). Cy5-labeled and unlabeled oligo DNA were annealed and used as the substrate. After reaction at 37°C for the indicated time points, the DNA was denatured and separated on a denaturing polyacrylamide gel (7 M urea) in 1×Tris–Borate–EDTA buffer. Fluorescent images were captured using the FUSION Chemiluminescence Imaging System with Spectral Capsule 480 and F535 filters (VILBER, France), and the signal intensities were quantified using ImageJ. Percentages of cleavage were calculated by dividing the band intensity of “Cut” by the total amount.

### Statistical analysis

The sample sizes and numbers of replicates are described in figures legends. Statistical analysis was performed using Student’s *t*-test or with GraphPad Prism version 9.3.2 for Macintosh (GraphPad Software). Multiple comparisons were performed by two-way analysis of variance (ANOVA). Data are represented as mean ± SD or the median as indicated.

## Results

### CARM1, a factor required for CPD removal in human cells, was identified by siRNA library screening

The incision step of GG-NER *in vitro* has been previously reconstituted using purified yeast proteins [33]. However, it is now known that histone modification enzymes, chromatin remodelers, or enzymes that modify factors essential for NER are also required for the smooth progression of GG-NER in mammalian cells [34–40]. Therefore, it is likely that as yet uncharacterized NER cofactors and modifications are required for NER progression. Therefore, we performed a screen using an siRNA library to identify new NER cofactors that have not yet been reported (Supplementary Fig. S1), using CPD removal as an indicator. The criterion for a positive siRNA in the screening was that CPD remained at 50% or more 24 h after UV irradiation at 12 J m^−2^, compared to cells treated with siXPC, an siRNA targeting XPC, a known factor essential for global genome NER. Screening was performed using siRNAs involved in DNA replication, ubiquitin, chromatin regulator, and transferase as known functions (Supplementary Fig. S1B). Screening was carried out duplicate in two sessions, the first with 622 independent siRNAs and the second with 496 independent siRNAs. The number of siRNAs with >50% in each screening was 21 in the first and 57 in the second (Supplementary Fig. S1C and Supplementary Table S1). The GG-NER cofactors identified by these screenings included a variety of proteins, among which CARM1 and METTL23 siRNAs reproducibly inhibited CPD removal. Since several lysine methyltransferases have been reported to be involved in NER, mainly via histone modification, but no arginine methyltransferase is known to be involved in NER, we investigated how CARM1 is required for GG-NER.

### The arginine methylation activity of CARM1 is required for CPD removal

**Figure 1.**
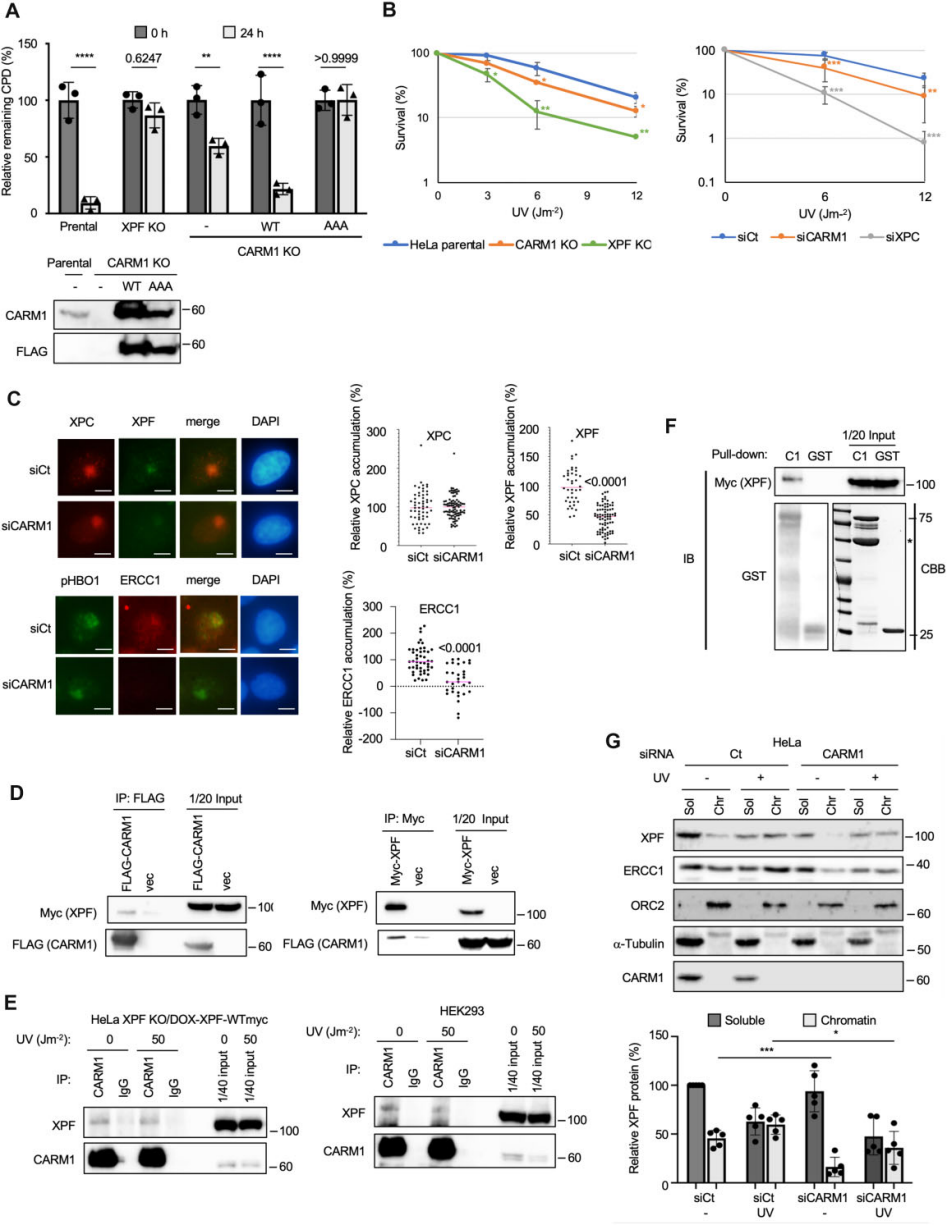
CARM1 deficiency suppresses XPF–ERCC1 accumulation at DNA damage sites and delays CPD removal. (**A**) CARM1 KO inhibited CPD removal ability. The defect of CPD removal was rescued by exogenous CARM1-WT but not by inactivated CARM1-AAA. Data are shown as the mean ± SD of biological three independent experiments (multiple comparisons: two-way ANOVA, **P < .01, ****P < .0001). (**B**) CARM1 KO and KD cells showed increased sensitivity to UV light. Biological three independent experiments were performed. Error bars represent SD *P < .05, **P < .01, and ***P < .001 (Student’s *t*-test). (**C**) Accumulation of XPC at local UV-exposed sites was not different between siCt and siCARM1, but XPF and ERCC1 accumulations were inhibited by siCARM1. Representative images of immunostaining for XPC and XPF and phosphor HBO1 and ERCC1 in local UV-irradiated HeLa cells. HeLa cells were irradiated with 50 J m^−2^ UV through an 8-μm pore membrane and fixed after 30 min of incubation. The mean intensity of the irradiated area was divided by the intensity of the non-irradiated area of the same nucleus and expressed as a relative intensity increase from the baseline (%). Scale bars, 5 μm. Data are shown as the mean (unpaired *t*-test). (**D**) CARM1 interacts with XPF in HEK293 cells. (**E**) XPF interacts with CARM1 in DOX-induced XPF stable expression cells and 293 cells. (**F**) XPF WT-Myc and GST-CARM1 interact in a GST pull-down assay. When XPF WT-Myc overexpressed in HEK293 cells was pulled down using GST-CARM1 expressed and purified in *E. coli*, XPF WT-Myc specifically co-precipitated. *An unknown *E. coli* protein that is co-purified when GST-CARM1 is purified from *E. coli*. (**G**) CARM1 KD cells showed decreased total protein content of XPF and decreased chromatin localization. XPF amounts were corrected for ORC2. The amount of XPF in each fraction is expressed as relative amount (%) to the siCt soluble fraction without UV. Biological five independent experiments were performed. Error bars represent SD. *P < .05 and ***P < .001 (Student’s *t*-test).

Since CARM1 depletion inhibited CPD removal, we tested whether this repair defect was dependent on CARM1 activity. To examine whether CARM1 is involved in CPD repair, we generated CARM1 KO HeLa cells (Supplementary Fig. S2A). CARM1 KO HeLa cells had a significant amount of CPD remaining in the nucleus 24 h after UV irradiation (Fig. 1A). This CPD removal defect was rescued by add-back of CARM1-WT, but not by add-back of an inactive CARM1 with a mutated *S*-adenosyl methionine binding site (CARM1-AAA). CARM1-AAA did not rescue the CPD removal activity, but rather increased the amount of CDP residues compared to CARM1 KO, possibly due to its effect as a competitive inhibition that also inhibits another protein arginine methyltransferase. One candidate, METTL23, is known to asymmetrically dimethylate histone H3R17 and has an overlapping substrate specificity with CARM1 [29]. To use the negative control for CPD removal, XPF KO HeLa cells were generated (Supplementary Fig. S2A). However, both CARM1 KO HeLa cells and CARM1 siRNA cells were still resistant to UV compared to XPF KO HeLa and siXPC cells, respectively. Both were more sensitive to UV irradiation than control cells (Fig. 1B), consistent with the fact that the loss of CARM1 methyltransferase activity due to CARM1 deficiency resulted in defective CPD removal. To identify which steps of GG-NER were inhibited by CARM1 depletion, we examined the accumulation of GG-NER factor at local DNA damage sites in the nucleus caused by local UV. The accumulation of XPC, which initially accumulates at UV damage sites, did not differ between control and siCARM1 cells, but the accumulation of XPF nuclease, which removes CPD, was significantly lower in siCARM1 cells (Fig. 1C). We also quantified the accumulation of ERCC1, a complex partner essential for XPF stability and nuclease activity, at the damage sites using phosphorylated HBO1 as a positive marker for local damage sites. In this study, ERCC1 accumulation at the damage sites was also suppressed in CARM1 knockdown (KD) cells (Fig. 1C). We also quantified the accumulation of XPF and ERCC1 at local UV damage sites in the METTL23 depleted cells. METTL23 is another arginine methyltransferase identified by the siRNA library screening. The depletion of this methyltransferase also suppressed the accumulation of XPF and ERCC1 (Supplementary Fig. S2B). To investigate whether XPF and CARM1 can physically interact, XPF-Myc, ERCC1, and CARM1-FLAG were co-expressed in HEK293 cells. The XPF-Myc binding signal was easily detected by immunoblotting with the Myc antibody after immunoprecipitation (IP) with the FLAG antibody. When IP–western blotting was performed with the reverse antibody combination, the CARM1-FLAG signal was also detected. These results suggest that CARM1 and XPF interact intracellularly (Fig. 1D and Supplementary Fig. S2C). We also examined the interaction of METTL23 with known NER factors by IP–western blotting. When METTL23 was overexpressed in HEK293 cells, it interacted with endogenous XPF (Supplementary Fig. S2D). These results suggest that CARM1 and METTL23 may act on XPF in a complementary manner and that the strong inhibition of CPD removal activity upon CARM1-AAA expression may be the result of competitive inhibition of METTL23. We then examined the interaction of stably expressed XPF and endogenous XPF with endogenous CARM1. XPF-Myc induced by DOX in HeLa cells and endogenous XPF in HEK293 cells were shown to co-precipitate with CARM1 (Fig. 1E). To confirm the binding between XPF and CARM1, we performed GST pull-down assays using GST-CARM1. GST-CARM1 specifically pulled down XPF from lysates containing XPF WT-Myc and ERCC1 were overexpressed in HEK293 cells (Fig. 1F). We next investigated whether the subcellular localization of XPF was altered by CARM1 depletion by biochemical cell fractionation (Fig. 1G). siCARM1 cells showed reduced chromatin localization of XPF in the presence and absence of UV irradiation. The total XPF protein content, the sum of the soluble fraction and chromatin fraction, was reduced in siCARM1 cells both before and after UV irradiation.

### Arginine sites of XPF are methylated in CARM1 overexpressing cells

**Figure 2.**
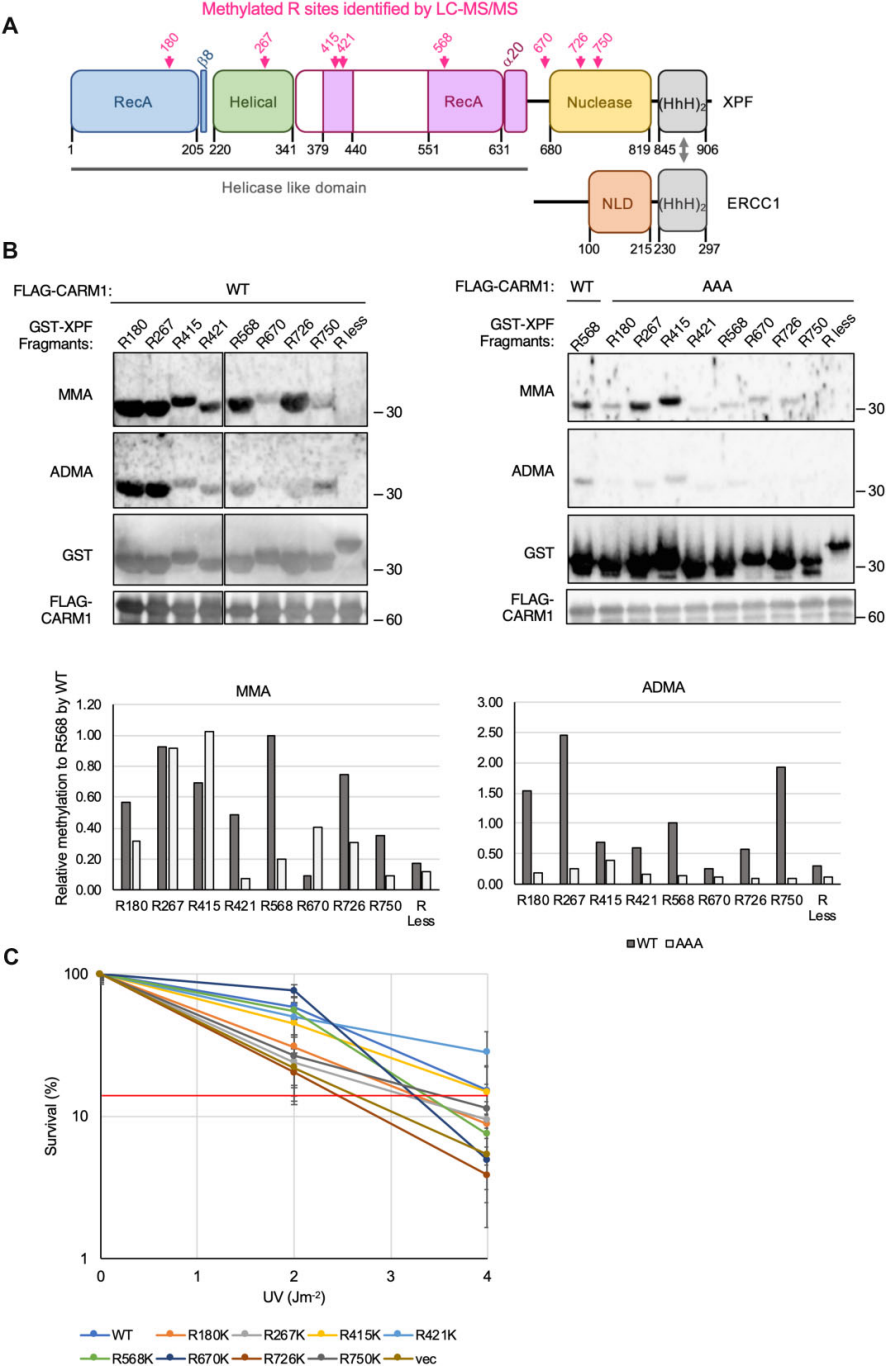
XPF is methylated at eight arginine sites in cell. (**A**) Schematic diagram of XPF–ERCC1 showing methylated arginine sites. Pink arrows indicate methylated arginine sites. (**B**) *In vitro* methylation assay with CARM1. AAA is CARM1 inactive mutant. MMA is monomethyl arginine and ADMA is asymmetric dimethyl arginine. The bar graph at the bottom shows the relative activity to the methylation activity at R568 of CARM1 WT. (**C**) Complementation of XP2YO-SV UV sensitivity by mutants in which the XPF arginine site to be methylated is replaced by lysine. XP2YO-SV are fibroblasts derived from XP-F patients. XP2YO-SV cells were transiently transfected with XPF WT and RK-Myc mutants. Cells were irradiated with indicated UV. Biological three independent experiments were performed. Error bars represent SD.

Since XPF binds to CARM1 intracellularly, we investigated whether XPF could be a substrate for CARM1. First, XPF-Myc was co-expressed with ERCC1 and CARM1-FLAG in HEK293 cells, and XPF-Myc was purified with Myc antibody. After digestion with Trypsin, methylated arginine sites were searched for using LC–MS/MS. Eight methylated arginine sites were identified: R180, R415, R421, R568, R670, R726, and R750 were monomethylated, and R267 was dimethylated (Supplementary Table S2). Of these, R180, R267, R415, R421, and R568 are located within the helicase-like domain from the XPF N-terminal to the central part. R726 and R750 on the C-terminal side are located in the nuclease domain. R670 is located in the linker between hyphen domain and nuclease domain (Fig. 2A). Similar to CARM1, when METTL23 was overexpressed in cells together with XPF–ERCC1, methylation sites in XPF were examined by LC–MS/MS, and monomethylation of the same arginine residues, except R267 and R670, was also identified (Supplementary Table S4).

*In vitro* methylation assays were performed to investigate whether these sites could be mono- and dimethylated by CARM1 (Fig. 2B). The peptides were expressed in *E. coli* as fusion of GST with 15 amino acids containing each arginine site identified by LC–MS/MS and purified as a substrate. As a negative control, a fusion of GST with 26 amino acids of XPF without the arginine moiety was used (R less, XPF amino acids 44–69). All XPF arginine sites tested were both monomethylated and asymmetrically dimethylated by CARM1, although the degree of methylation varied (Fig. 2B). In contrast, no CARM1 methylation signal was detected in the negative control GST-R less. As another negative control, a methylation assay was performed using CARM1 AAA, an inactive form of CARM1. The activities of WT and AAA were normalized using methylation of R568 by WT. Surprisingly, monomethylations of R267 and R415 by CARM1 AAA were comparable to that of WT. In contrast, dimethylation by CARM1 WT was greater than that by AAA at all R sites tested. This suggests that the monomethylation activity of R267 and R415 may be due to an unknown methyltransferase that contaminates CARM1 during immunoprecipitation. The methylation activity of WT and AAA is shown in the lower graph of Fig. 2B. The effect of these CARM1-methylated arginine sites on XPF-dependent UV tolerance was investigated using a clonogenic assay. Cells were XP2YO-SV cells established from XP-F patients and tested for UV sensitivity by transiently transfection with XPF WT-Myc or XPF-Myc whose arginine sites were replaced by lysine (Fig. 2C). The six arginine sites that showed stronger UV sensitivity than WT at the 4 J m^−2^ irradiation were R180K, R267K, R568K, R670K, R726K, and R750K (mutants with lower survival rates than the red line in UV 4 J m^−2^ irradiation).

### UV-sensitive six arginines are required for CPD removal and chromatin localization

**Figure 3.**
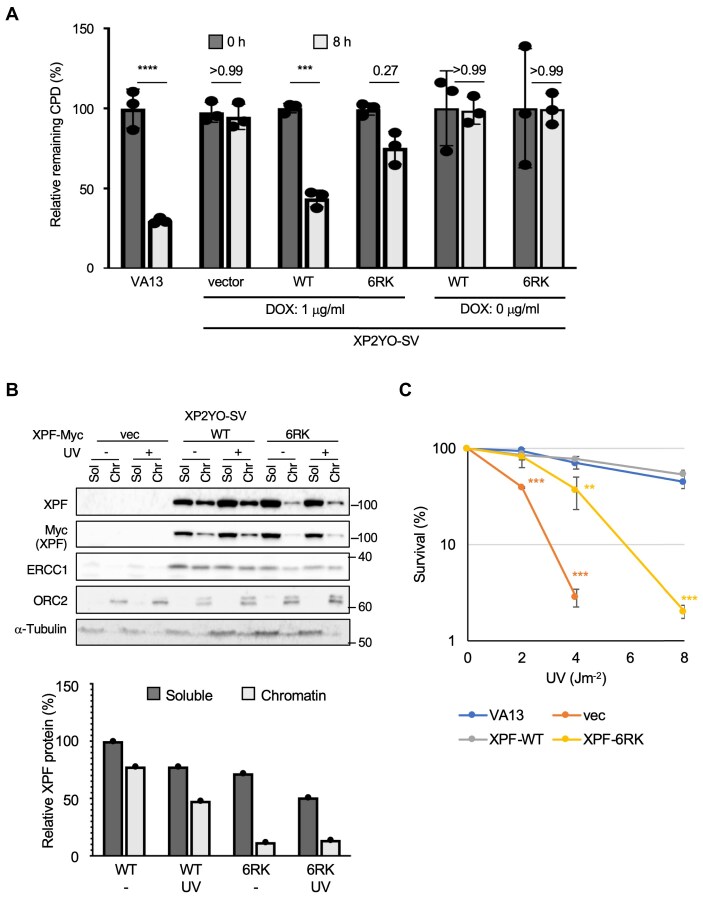
XPF 6RK with six arginine sites replaced by lysine showed defects in CPD removal, subcellular localization, and UV sensitivity. (**A**) DOX-inducible XPF WT complemented the CPD removal ability of XP2YO-SV, but XPF 6RK was partially deficient in complementation. VA13 is a normal human-derived fibroblast. Data are shown as the mean ± SD of biological three independent experiments (multiple comparisons: two-way ANOVA, ***P < .001, ****P < .0001). (**B**) XPF 6RK expressing cells showed decreased total protein content of XPF and decreased chromatin localization. (**C**) XPF 6RK expressing cells showed increased sensitivity to UV light. Biological three independent experiments were performed. Error bars represent SD. **P < .01, ***P < .001 (Student’s *t*-test).

Transient transfected cells are difficult to control XPF expression levels between experiments to the same extent as endogenous XPF, so XPF WT-Myc and XPF 6RK-Myc (R180K, R267K, R568K, R670 K, R726K, and R750K were all replaced with lysine) were expressed in DOX-inducible stable transformant cells. When a CPD removal assay was performed using these cells, the amount of residual CPD in the positive control, VA13 cells derived from normal human fibroblasts, decreased to <30% within 8 h after UV irradiation. Transfected empty vector did not remove any CPD (Fig. 3A). In XPF WT-Myc expression-induced cells with DOX, CPD removal progressed to 40% residual CPD, whereas in XPF 6RK, CPD removal was delayed and ∼80% of CPD remained (Fig. 3A). The subcellular localization of these DOX-inducible XPF cells in the presence or absence of UV damage showed that XPF 6RK-Myc localized to chromatin in a reduced amount regardless of UV damage (Fig. 3B). Consistent with the results of CPD removal capacity, XPF 6RK showed a significant increase in sensitivity to UV, although not to the same extent as in vector controls (Fig. 3C).

### Predicting the potential impact of CARM1-dependent methylated arginine residues

**Figure 4.**
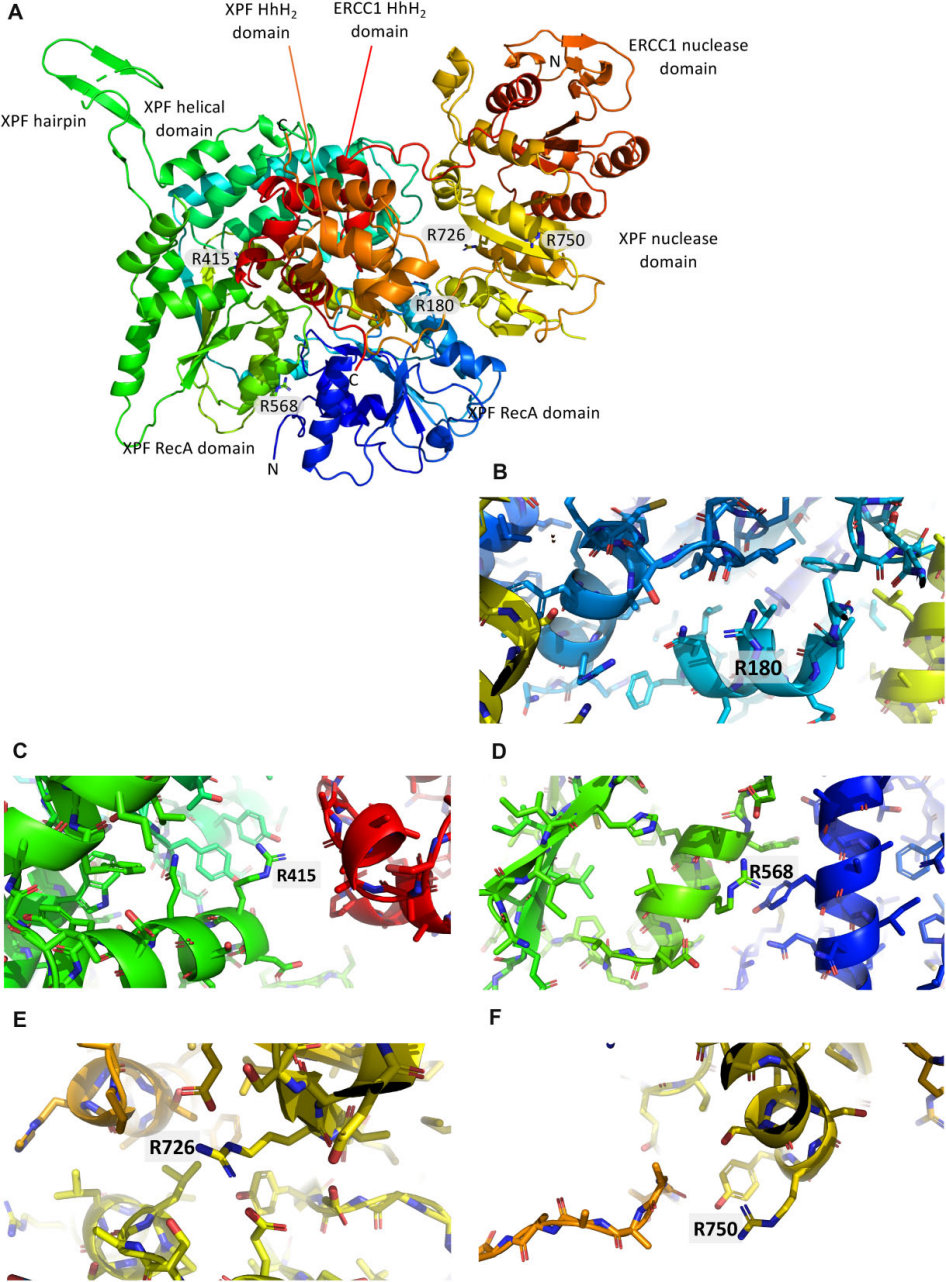
Mapping CARM1-dependent arginine methylation sites onto the human XPF–ERCC1 structure. (**A**) Overall structure of the auto-inhibited XPF–ERCC1 heterodimer (PDB code 6SXA). Structure is shown as a cartoon using rainbow colors for XPF from the N-terminus to C-terminus highlighting the first RecA domain (blue) and second RecA domain (green) the helical domain (green/cyan), the nuclease domain (yellow), and the HhH_2_ domain (red). ERCC1 is colored orange for both its central domain and HhH_2_ domain. This figure was prepared using PyMol. (**B**–**F**) Close-up views of the structural environment surrounding each of the five putative methylated arginine sites identified as targeted by CARM1. Colors are as described for panel (A).

Five of the arginine residues identified by LC–MS/MS were suggested to be CARM1-dependent at R180, R415, R568, R726, and R750 (Supplementary Table S3). The potential role of these arginine residues to alter the conformation of XPF–ERCC1 nuclease was predicted by mapping them onto a recent cryo-EM structure of full-length XPF–ERCC1 in an auto-inhibited state [Protein Data Base (PDB) code 6SXA; Fig. 4A–F]. Structurally, XPF has two divergent RecA domains within its N-terminal helicase-like domain preceding a central nuclease domain and an HhH_2_ domain at the C-terminus (Fig. 4A). The evolutionarily related ERCC1 has a nuclease-like central domain followed by an HhH_2_ domain, which together engage XPF through its nuclease domain and HhH_2_ domain [15]. The R180 side chain, within the first RecA domain of XPF, forms a hydrogen bond with the main chain of L140 carbonyl and S142 side chain, so is likely structurally important (Fig. 4B). R415 side chain within the second RecA domain forms a hydrogen-bond to A272 main chain carbonyl of ERCC1 HhH_2_ domain and hydroxyl of Y297 of XPF helical domain. Alteration may impact on the auto-inhibited state of XPF–ERCC1 (Fig. 4C). R568 side chain forms a salt bridge with D562 on the surface of a key highly charged helix within the second RecA fold of XPF (Fig. 4D). It may well be therefore structurally important to the contacts close to the RecA–RecA domain interface. Disruption by a lysine would preserve the formal positive charge but would may disrupt the folding of this region explaining the reduced expression. R726 side chain from the nuclease domain forms an intricate double salt bridge to side chains of both E760 and E690, this side chain is highly conserved and involved in substrate recognition (Fig. 4E). R750 side chain pi-stacks on Y751 aromatic ring and hydrogen-bonds to its hydroxyl. This hydroxyl in turn couples to the XPF linker region carbonyl of T832 in between the catalytic nuclease domain and HhH_2_ hairpin domain (Fig. 4F). Thus, several arginines identified as modified by methylation appear to play important structural and functional roles within the XPF–ERCC1 heterodimer.

### Replacement of XPF arginine 568 with lysine results in defective CPD removal and sensitivity to UV

**Figure 5.**
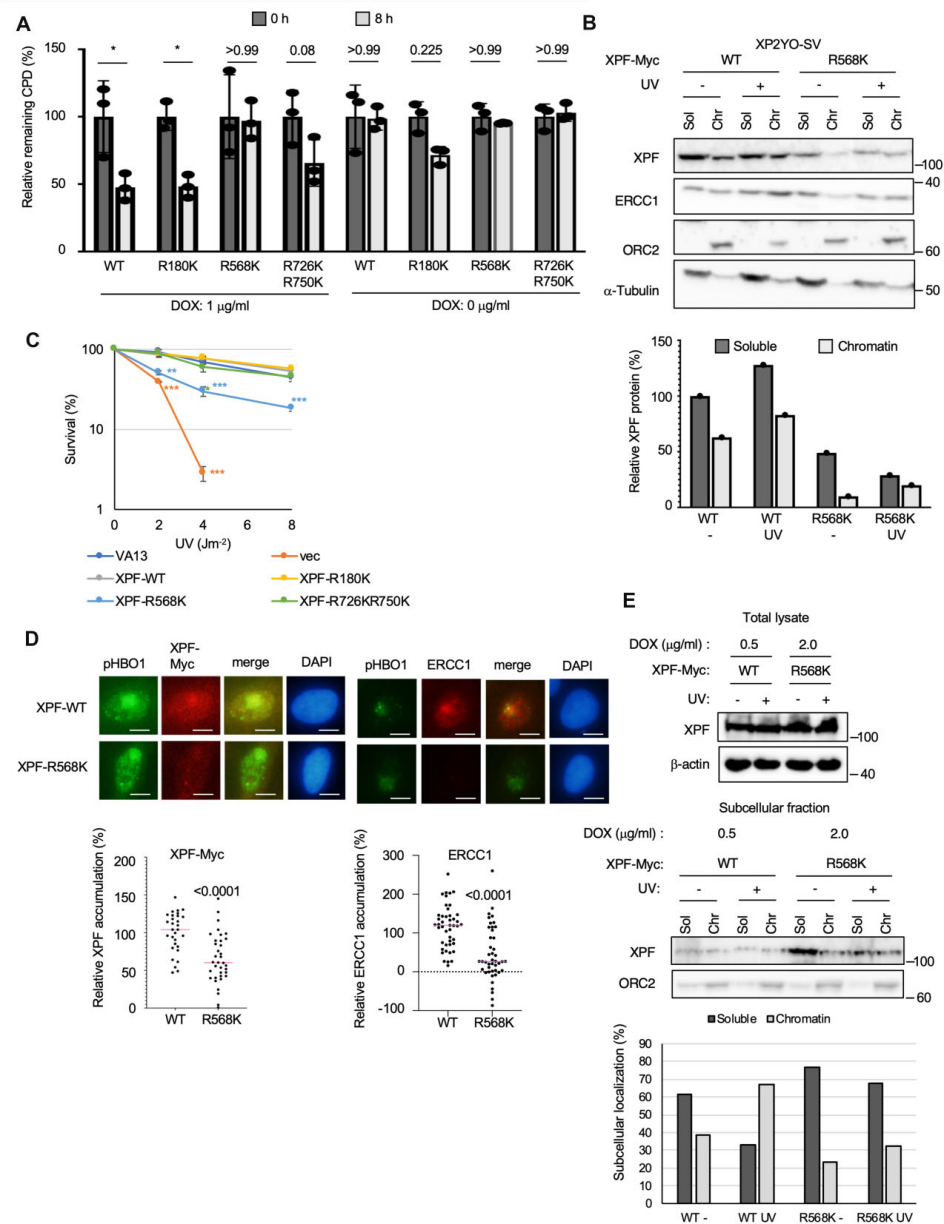
XPF R568K with R568 arginine site replaced by lysine showed defects in CPD removal, subcellular localization, UV sensitivity, and accumulation at local UV sites. (**A**) DOX-inducible XPF WT and R180K complemented the CPD removal ability of XP2YO-SV, but XPF R568K was completely deficient, and R726KR750K were partially deficient in complementation. Data are shown as the mean ± SD of biological three independent experiments (multiple comparisons: two-way ANOVA, *P < .05). (**B**) XPF R568K expressing cells showed decreased total protein content of XPF and decreased chromatin localization. (**C**) XPF R568K expressing cells showed increased sensitivity to UV light. Biological three independent experiments were performed. Error bars represent SD. **P < .01, ***P < .001 (Student’s *t*-test). (**D**) Accumulation of XPF R568K and ERCC1 at local UV sites was inhibited in R568K expressing HeLa cells. Representative images of immunostaining for pHBO1 and XPF-Myc and pHBO1 and ERCC1 in local UV-irradiated HeLa cells. HeLa cells were irradiated with 50 J m^−2^ UV through an 8-μm pore membrane and fixed after 30 min of incubation. Scale bars, 5 μm. Data are shown as the mean (unpaired *t*-test). (**E**) In cells expressing XPF R568K to the same extent as WT, XPF R568K-Myc still showed a reduced amount of chromatin bound.

**Figure 6.**
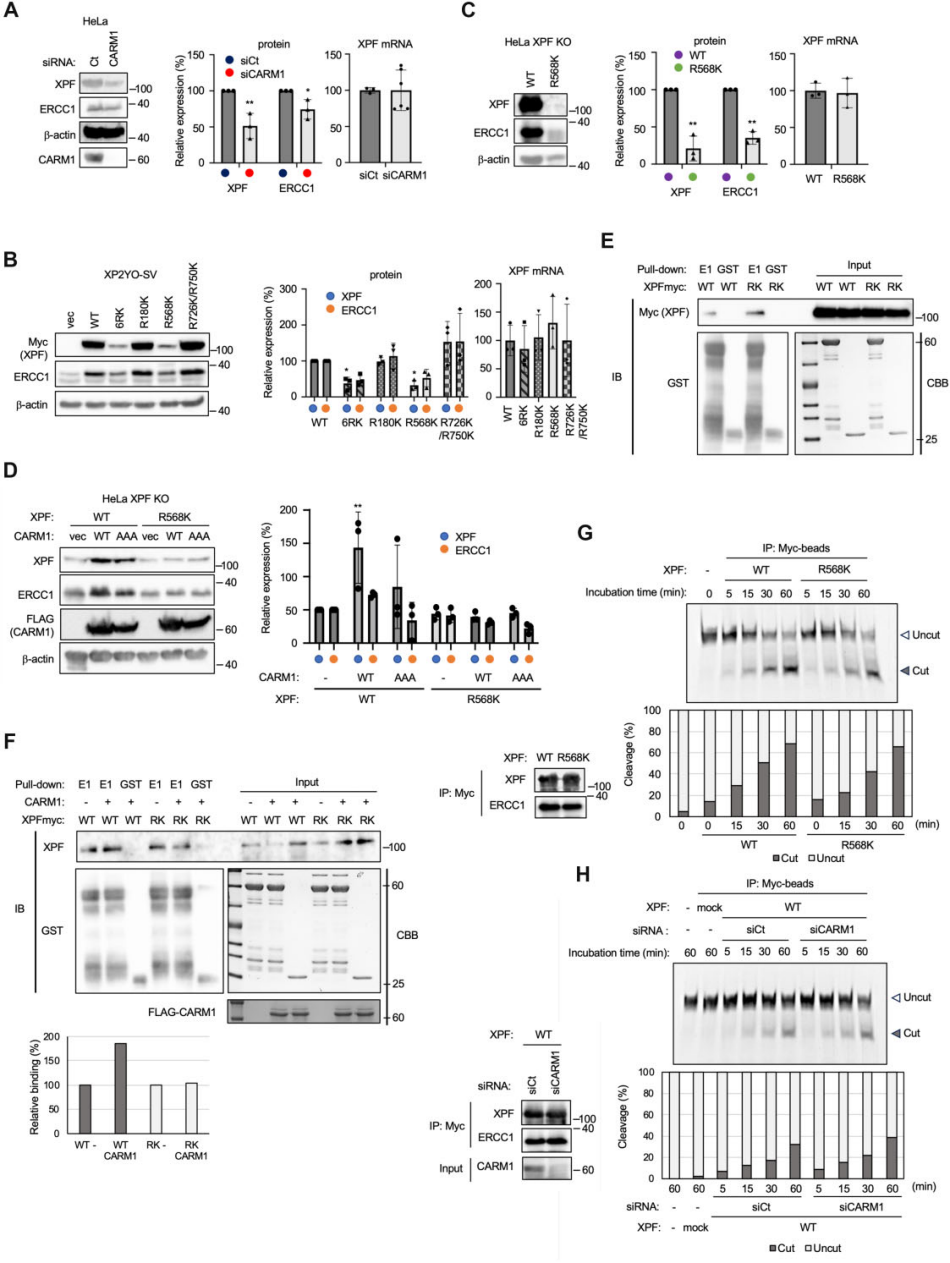
XPF in CARM1 depleted cells and DOX-induced XPF 6RK and R568K show no change in messenger RNA (mRNA) expression but a marked decrease in protein levels. (**A**) XPF and ERCC1 proteins in CARM1 depleted cells were significantly reduced. (multiple comparisons: two-way ANOVA, *P < .05, **P< .01). (**B**) DOX-inducible XPF 6RK and R568K in XP2YO-SV cells had significantly reduced expression of XPF and ERCC1 proteins, although mRNA expression was unchanged. (Multiple comparisons: two-way ANOVA, *P < .05). (**C**) DOX-inducible XPF R568K in HeLa cells had significantly reduced expression of XPF and ERCC1 proteins, although mRNA expression was unchanged (multiple comparisons: two-way ANOVA, **P< .01). (**D**) XPF and ERCC1 protein expression in DOX-inducible XPF WT or R568K HeLa cells overexpressing CARM1-WT or AAA in XPF (multiple comparisons: two-way ANOVA, **P< .01). (**E**) GST pull-down assay using *in vitro* translated XPF WT and R568K and GST-ERCC1 expressed and purified in *E. coli*. E1 is ERCC1, RK is XPF R568K-Myc. (**F**) XPF, which had been subjected to an *in vitro* methylation assay using affinity-purified CARM, was pulled down using GST-ERCC1. XPF was *in vitro* translated and affinity purified using MycHis-tag. CARM1 was overexpressed in HEK293 cells and affinity purified using FLAG-tag. GST-ERCC1 was expressed in *E. coli* and purified using glutathione beads. The bar graph shows the relative binding value against WT (WT−) that was not subjected to the methylation assay. Relative binding of XPF was corrected with GST-ERCC1. (**G**) Nuclease assay using XPF WT and R568K. XPF-Myc-ERCC1 overexpressed in HEK293 cells was affinity purified with anti-Myc-tag mAb magnetic beads and then incubated with fluorescently labeled oligo DNA substrate for the indicated time. (**H**) siCt and siCARM1 cells were co-expressed with XPF WT-Myc-ERCC1 and affinity purified, followed by nuclease assays. The assays were incubated for the indicated times. Mock sample was prepared using anti-Myc-tag mAb magnetic beads incubated with mock transfected HEK293 cells.

Four CARM1-dependently methylated arginine sites (R180, R568, R726, and R750) were replaced with lysine (R180K, R568K, and R726KR750K) and induced expression in XP2YO-SV cells in a DOX-inducible manner. When the CPD removal ability of these cells was examined, a significant defect was observed in XPF R568K (Fig. 5A). Localization of overexpressed XPF R568K revealed a more diffused signal in the cytoplasm compared to other RK mutants (Supplementary Fig. S3A). Such a phenotype has also been observed in XPF mutants in XP-F patients [23]. Subcellular localization of XPF R568K-Myc was compared with that of XPF WT-Myc, and as shown for XPF 6RK-Myc, localization of XPF R568K-Myc was reduced in the chromatin with or without UV damage (Fig. 5B). The total amount of XPF was also found to be reduced compared to WT (Fig. 5B). Consistent with a decrease in CPD removal, XPF R568K-Myc expressing cells showed an increase in UV sensitivity (Fig. 5C). To investigate the accumulation of XPF R568K-Myc at local UV sites, DOX-inducible XPF WT-Myc and R568K-Myc stable transfected cells were established in XPF KO HeLa cells. When UV sensitivity was examined using these cells, cells expressing XPF R568K-Myc were UV-sensitive, as was the case when mutant-XPF was induced in XP2YO-SV cells (Supplementary Fig. S3B). Consistent with reports that XPF–ERCC1 is an essential nuclease not only for NER but also for ICL repair, XPF R568K expressing HeLa cells were sensitive to the drug MMC, which induces DNA interstrand crosslinks (Supplementary Fig. S3C). XPF R568K-Myc HeLa cells showed significantly lower accumulation of XPF at local UV sites than XPF WT-Myc HeLa cells (Fig. 5D). On the other hand, after UV irradiation, phospho S50 and S53 of HBO1 (pHBO1), which localizes to DNA damage sites in a DDB2-dependent manner, accumulated at the same amount of damage sites. We also examined the accumulation of ERCC1, an essential partner of the XPF function, in these cells. Consistent with the reduced accumulation of XPF R568K-Myc at the damage sites, ERCC1 accumulation was also suppressed in XPF R568K-Myc expressing cells (Fig. 5D). All experiments using XPF induced by DOX so far were performed under conditions in which XPF expression was induced with 1 μg/ml DOX for 24 h. At that time, the protein expression level of the XPF R568K-Myc mutant was reduced to ∼30% of that of the WT (Fig. 6B). Therefore, the phenotype of the R568K mutant is primarily due to differences in protein levels caused by a lack of protein stability. To address whether the phenotype would change when the R568K protein expression level was similar to that of the WT, we performed an experiment in which the expression levels of the WT and R568K were comparable. In Fig. 5E, the amount of DOX added was 0.5 μg/ml for the WT and 2.0 μg/ml for the R568K to induce the same level of protein expression, and subcellular fractionation was performed. Comparing the percentage of XPF protein present in the soluble and chromatin bounds under each condition, we found that the amount present in the chromatin bound of R568K was reduced compared to that in the WT, both with and without UV irradiation. This suggests that R568K has a reduced chromatin-binding ability, even though the protein expression level is the same.

### XPF protein expression is reduced in CARM1 depleted HeLa cells and XPF R568K expressing XP2YO-SV and HeLa cells

Decreased XPF protein expression was suspected to be responsible for the reduced CPD removal capacity caused by CARM1 loss. Therefore, the protein and mRNA levels of XPF expressed in siCARM1 cells, XPF RK mutants expressed in XP2YO-SV cells, and R568K-Myc expressed in HeLa cells were quantified and compared to XPF and XPF WT-Myc in control cells (Fig. 6A–C). XPF protein in CARM1 depleted cells was reduced to ∼50% of XPF in control cells (Fig. 6A). However, the amount of XPF mRNA was the same in control cells and CARM1 depleted cells, suggesting that the stability of the protein was lost in CARM1 depleted cells. It is well known that XPF and ERCC1 proteins require a mutually stabilizing interaction with each other after being translated. Therefore, the amount of ERCC1 protein was also quantified and found to be decreased in CARM1 depleted cells, similar to XPF (Fig. 6A). Comparing the protein levels of XPF and RK mutants expressed DOX-inducibly in XP2YO-SV cells, it was found that 6RK and R568K were significantly downregulated (Fig. 6B). In agreement with this decreased XPF expression, the expression level of ERCC1 protein was also reduced. In contrast, all XPF-Myc mRNA expression levels remained unchanged, suggesting that XPF 6RK and R568K cannot interact with ERCC1 because they are unable to fold into the correct conformation, leading to a reduction in protein levels. As seen in the XP2YO-SV cells, XPF R568K-Myc and ERCC1 protein levels in HeLa cells are reduced compared to WT; however, mRNA expression levels in XPF WT and R568K cells remained unchanged (Fig. 6C). XPF in CARM1 depleted cell and XPF R568K-Myc showed a loss of protein stability. Conversely, we investigated whether XPF protein stability increases when CARM1 is overexpressed and whether CARM1 methylation activity is required for this (Fig. 6D), using XPF WT-Myc and XPF R568K-Myc expressing cells. The overexpression of CARM1 WT was found to increase the expression level of XPF WT protein. The overexpression of the CARM1 methylation inactive mutant, CARM1-AAA, also increased the expression level of XPF WT compared to the vector control, although the effect was less than that observed for CARM1-WT. This effect of CARM1-WT and AAA on XPF expression was not observed for XPF R568K mutant. The effect of CARM1 overexpression on protein stability was less pronounced for ERCC1. In particular, the trend of increased expression of ERCC1 by CARM1-AAA was limited to a smaller extent (Fig. 6D). We confirmed by *in vitro* binding assays that the XPF R568K-Myc mutant did not lose its ability to bind to ERCC1 due to the single amino acid substitution. *In vitro* translated XPF WT-Myc and R568K-Myc were pulled down by purified GST-ERCC1 expressed in *E. coli* (Fig. 6E). We further examined whether the *in vitro* translated XPF WT-Myc and R568K-Myc mutants were altered in their ability to bind to GST-ERCC1 after an *in vitro* methylation assay with CARM1 (Supplementary Fig. S4). After CARM1-mediated methylation, XPF WT-Myc increased its ability to bind GST-ERCC1, whereas the XPF R568K-Myc mutant showed no change in its binding ability (Fig. 6F). These data demonstrated that methylation of XPF by CARM1 promotes its binding to ERCC1. We further investigated whether this methylation affected XPF–ERCC1 nuclease activity. First, XPF WT-Myc-ERCC1 and R568K-Myc-ERCC1 were affinity-purified and subjected to *in vitro* nuclease assay. When equivalent molar concentrations of XPF were used to detect nuclease reactions over time, the WT and RK showed almost the same activity (Fig. 6G). Next, XPF WT-Myc-ERCC1 expression in CARM1 depleted cells was compared with that of the control (siCt) (Fig. 6H). There was no difference in the nuclease activity in this assay. The negative control (mock-IP) showed no nuclease activity, demonstrating that this nuclease activity was due to XPF–ERCC1.

In summary, several arginine sites in XPF are monomethylated by CARM1. Pull-down assays using *in vitro* translated XPF WT and R568K showed no difference in the binding ability to ERCC1. It was also suggested that XPF’s binding of XPF to ERCC1 is promoted when R568 is methylated by CARM1 before binding to ERCC1. It has also been suggested that R568 methylation by CARM1 promotes XPF binding to the chromatin. Thus, methylation by CARM1 is necessary in cells to respond to DNA damage caused by UV irradiation, to supply sufficient XPF–ERCC1, and to localize to chromatin and promote repair.

## Discussion

XPF–ERCC1 is a well-conserved structure-specific endonuclease that promotes DNA repair by nicking double-strand DNA in structure-specific substrates within DSB repair and ICL repair as well as NER. Structurally, XPF has an N-terminal helicase-like domain preceding a central nuclease domain and an HhH_2_ domain at the C-terminus. ERCC1 is thought to share the same evolutionary origin as XPF and has a similar structure. ERCC1 has an HhH_2_ domain at its C-terminus and a nuclease-like domain at its N-terminus. XPF and ERCC1 interact with each other through their respective nuclease/nuclease-like domains as well as through their HhH_2_ domains. As XPF–ERCC1 has been shown to be a nuclease that contributes to drug resistance to platinum drugs such as cisplatin, it is important from a clinical point of view to clarify the regulatory mechanism of its activity. In this study, we demonstrated that the protein arginine methyltransferase, CARM1, is required for maintaining the stability of XPF–ERCC1.

The PRMT family of enzymes exhibit a catalytic activity to add a methyl group from SAM to the guanidino group of the arginine side chain. Nine structurally similar PRMT family enzymes have been found in mammalian cells and there are three types of methylation: Type I (EC 2.1.1.319), which catalyzes MMA and ADMA of the guanidino group in the arginine side chain; Type II (EC 2.1.1.320), which catalyzes MMA and SDMA; and Type III, which catalyzes only MMA. CARM1 belongs to Type I and catalyzes MMA and ADMA. PRMT1, which belongs to Type I like CARM1, activates MRE11 nuclease activity by ADMA in DNA damage repair [41, 27]. On the other hand, PRMT5, which belongs to Type II, stabilizes p53 binding protein (53BP1) by SDMA [42]. Thus, two different repair pathways (homologous recombination and non-homologous end joining) of DNA DSB are regulated by through different arginine methylation. Although there have been no reports of CARM1 being directly involved in DNA damage repair, we report here for the first time that CARM1 interacts with XPF and contributes to the stability of the XPF–ERCC1 complex, thereby facilitating NER. We predicted that CARM1 methylated XPF as a substrate because CARM1 physically interacts with XPF intracellularly and CARM1 depleted cells show significantly inferior accumulation of XPF at the site of damage after UV irradiation. Since many arginine residues have been found among the mutated amino acids identified in patients with XP-F complement group, we considered the possibility that defective methylation by CARM1 may contribute to the XP-F complement group and identified XPF methylated arginine sites by LC–MS/MS. Contrary to expectations, no methylation could be found at the arginine sites that were mutated to other amino acids in the XP-F complement group, but eight arginine sites were found to be targeted for methylation. Among these, the five reduced methylation sites (R180, R415, R568, R726, and R750) in CARM1 depleted cells were located at sites that could affect structural maintenance when mapped onto the cryo-EM XPF–ERCC1 structure. Studies using DOX-inducible XPF R568K expressing XP2YO-SV cells showed that of these arginine sites, R568 is the one that most affects XPF protein stability. Replacement of this site by lysine results in the loss of XPF protein stability maintenance by CARM1, although CARM1 inactive mutants (CARM1-AAA) also show partial XPF protein stability maintenance activity in experiments in which CARM1 is overexpressed. This activity was not observed for XPF R568K, suggesting that R568 is not only a substrate for CARM1 but also an amino acid that is very important for the XPF-binding ability of CARM1. The increased expression of XPF protein by CARM1-WT resulted in increased expression of ERCC1 protein, whereas this effect was less pronounced in CARM1-AAA, suggesting that methylation of R568 by CARM1 facilitates complex formation with ERCC1. CARM1-AAA contributes to the stabilization of XPF protein to some extent, whereas the CPD removal activity is significantly inhibited when CARM1-AAA is added back to CARM1 KO cells, which is a contradictory result. These seemingly contradictory results may suggest that other methyltransferases that methylate XPF also work redundantly and that the CARM1 AAA mutant may have a dominant negative effect on them. In fact, another arginine methyltransferase, METTL23, has been identified in a screen for CPD removal.

In this study, we performed *in vitro* GST pull-down experiments and showed that the R568K-Myc mutant had the same ERCC1 binding ability as the WT-Myc, and that, although XPF WT-Myc increased its binding ability to ERCC1 after *in vitro* methylation by CARM1, the R568K-Myc mutant did not. These results suggest that XPF, after translation in cells, is enhanced in binding to ERCC1 by methylation of R568 by CARM1. We also demonstrated that there was no difference in nuclease activity between XPF WT and R568K, or between XPF–ERCC1 in CARM1 depleted cells and those in control cells. We also showed that the chromatin bound amount of the R568K-Myc mutant was reduced even when the amount of DOX was increased to maintain the same protein expression level as the WT-Myc. The reason for this reduction in chromatin-bound amount is unclear. Collectively, these results suggest that the inhibition of XPF–ERCC1 function in CARM1 depleted cells is due to the suppression of XPF–ERCC1 binding as well as impaired chromatin localization, and not due to a defect in nuclease activity. There have been other reports of methylation of arginine residues in non-histone proteins affecting subcellular localization. For example, PRMT6, a type I PRMT similar to CARM1, methylates chromatin condensation 1 (RCC1) to increase chromatin-bound RCC1 and enhance its mitotic activity [43]. One possibility is that the helicase-like domain in which XPF R568 is located contains an RPA binding region that required to localizes to the CPD after UV irradiation. Methylation of R568 may have an effect that favors RPA binding. Whether monomethylation of R568 is induced in the activated state of XPF after UV irradiation remains to be investigated in the future. It also remains to be investigated whether there is a reader protein that recognizes XPF R568 methylation and how it relates to the chromatin localization of XPF–ERCC1. Further studies are needed to investigate the possibility that CARM1 and METTL23 are involved in genetic diseases caused by lack of XPF activity and to approach anticancer drug resistance from the perspective of CARM1 and METTL23.

## Supplementary Material

gkaf355_Supplemental_Files

## Data Availability

The data generated in this study are available upon request from the corresponding author.
