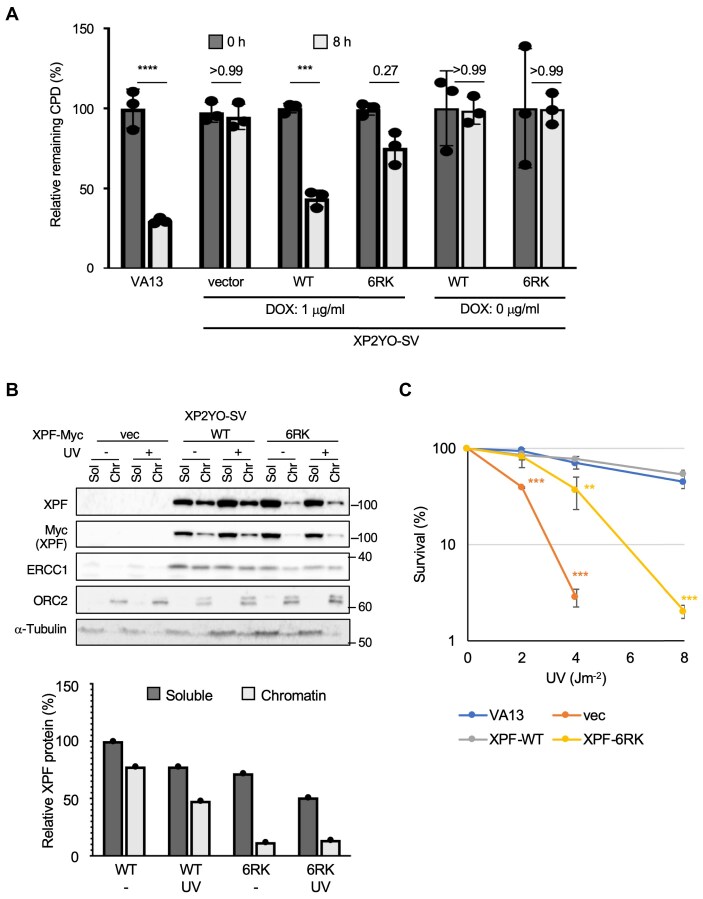# Correction to ‘CARM1/PRMT4 facilitates XPF–ERCC1 heterodimer assembly and maintains nucleotide excision repair activity’

**DOI:** 10.1093/nar/gkaf490

**Published:** 2025-05-30

This is a correction to: Hiroyuki Niida, Masahiko Ito, Kenta Iijima, Akira Motegi, Rin Ogihara, Hironobu Akiyama, Chiharu Uchida, Satoshi Sakai, Tatsuya Ohhata, Atsushi Hatano, Michiko Hirose, Atsuo Ogura, Masaki Matsumoto, Neil Q. McDonald, Masatoshi Kitagawa, CARM1/PRMT4 facilitates XPF-ERCC1 heterodimer assembly and maintains nucleotide excision repair activity, *Nucleic Acids Research*, Volume 53, Issue 8, 8 May 2025, gkaf355, https://doi.org/10.1093/nar/gkaf355

During the preparation of the accepted manuscript for publication, Figures 5A, 5B, and 5C were inadvertently inserted in place of Figures 3A, 3B, and 3C. This error has been rectified by replacing these panels with the correct original versions intended for Figure 3.

This change does not affect the results, discussion and conclusions presented in the article. The published article has been updated.