# Supplementary material for: CARM1/PRMT4 facilitates XPF–ERCC1 heterodimer assembly and maintains nucleotide excision repair activity
Source: Nucleic Acids Res. 2025 Apr 30;53(8):gkaf355. doi: 10.1093/nar/gkaf355 (PMC12041854; doi:10.1093/nar/gkaf355)

Supplementary figure legends

Supplementary Fig. 1 Summary of siRNA library screening to identify intracellular molecules required for GG-NER

(A) Strategy of the library screening. (B) Types of siRNA used in siRNA library screening. (C) Distribution of screening results from lot.1 (Left) and lot.2 (Right) experiments.

Supplementary Fig.2A Genomic sequences of CARM1 and XPF KO HeLa cells. (B) Accumulation of XPF at local UV sites were inhibited in METTL23 KO MEF. Representative images of immunostaining for XPC and ERCC1 in local UV-irradiated cells. Cells were irradiated with 50 Jm^{-2} UV through an 8-μm pore membrane and fixed after 30 min of incubation. Scale bars, 5 μm. Data are shown as the mean (Unpaired t-test). Deficiency of METTL23 arginine methyltransferase results in defective accumulation of XPF at local UV sites. (C) Binding assay of XPF-Myc, ERCC1 and FLAG-CARM1 overexpressed in 293 cells.(D) Binding study of METTL23 and GG-NER factors in HeLa cells overexpressing mouse METTL23.

Supplementary Fig. 3A Intracellular distribution of a mutant in which the methylated arginine moiety is replaced by lysine in HeLa cells upon transient transfection. Scale bars, 20 μm. The number of cells in which XPF was diffused in the cytoplasm was shown in the graph as a percentage of the total number of cells. (B) UV and (C) MMC sensitivity of XPF KO HeLa cells expressing DOX-inducible XPF WT or R568K. Biological three independent experiments were performed. Error bars represent S.D.

Supplementary Fig. 4 In vitro methylation assay of XPF by CARM1. MMA is mono-methyl arginine. XPF was affinity purified using Myc antibody after in vitro translation. CARM1 was overexpressed in 293 cells and affinity purified using FLAG antibody. In the methylation assay, XPF was incubated at 30°C for 3 hours in a reaction solution containing CARM1 and 500 μM SAM.

Supplementary table legend

Supplementary Table 1 Summary of siRNA library screening. Targets with CPD residuals greater than 50% compared to siXPC were considered as hits.

Supplementary Table 2 Peptide summary, fragment matches and fragment spectrum of methylated arginine fragments identified by LC-MS/MS analysis.

Supplementary Table 3 LC-MS/MS analysis was used to identify arginine moieties that are CARM1-dependent methylated. Cells were collected 30 min after irradiation with UV 0 Jm^{-2} or 50 Jm^{-2}.

Supplementary Table 4 The arginine sites of XPF identified when METTL23 was overexpressed together with XPF-Myc and ERCC1 were identified by LC-MS/MS.

Niida et al. supplementary Fig. 1

A

HeLa cell
↓
Transfect siRNA library
↓
UV 15 Jm^{-2}
↓
Incubate at 37℃ for 24 hr
↓
Immunostaining with anti-CPD antibody
(Green fluorescence)
↓
Measure intensities of green fluorescence

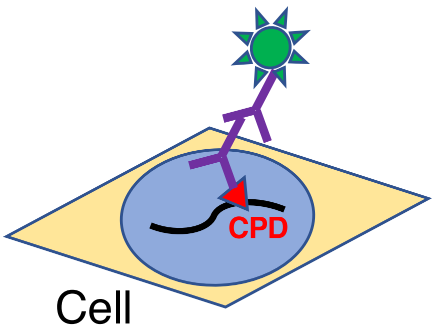

- Criteria of positive siRNA in the screening

$$\frac{\left[\dfrac{Target^{CPD}/Target^{DAPI}}{Ct^{CPD}/Ct^{DAPI}} - 1\right]}{\left[\dfrac{XPC^{CPD}/XPC^{DAPI}}{Ct^{CPD}/Ct^{DAPI}} - 1\right]} \times 100 = > 50$$

B

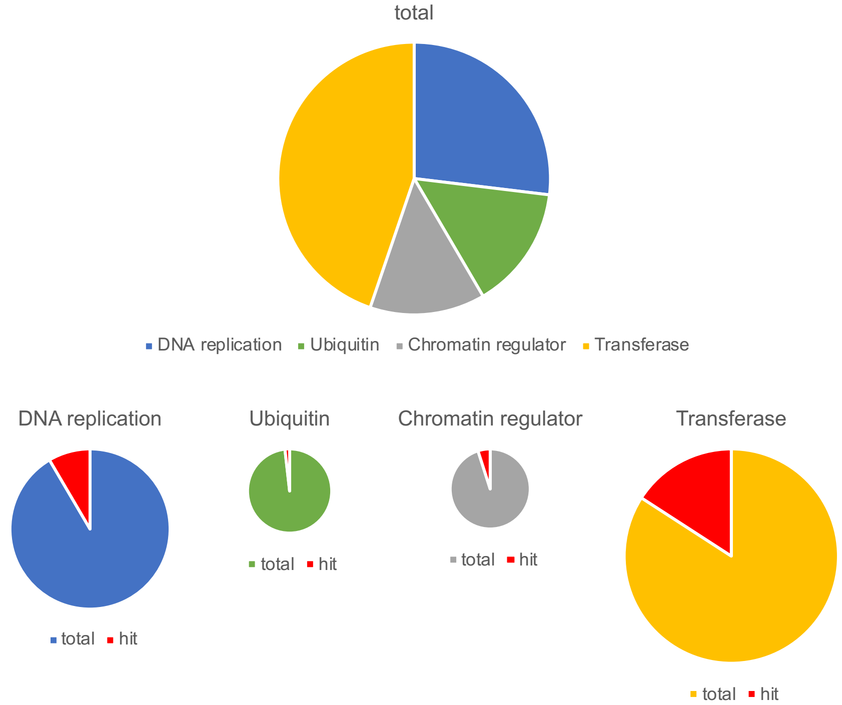

C

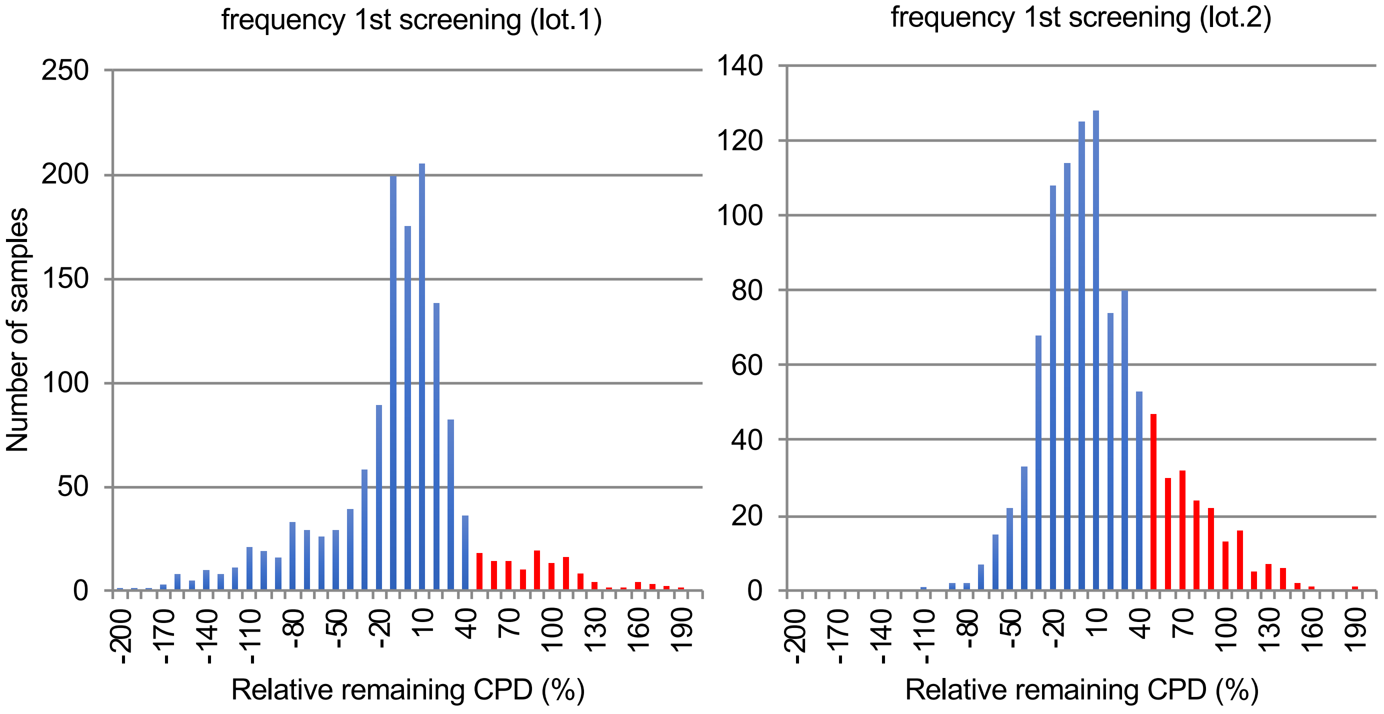

A

Niida et al. supplementary Fig.2

Genomic sequence of CARM1 KO HeLa cell

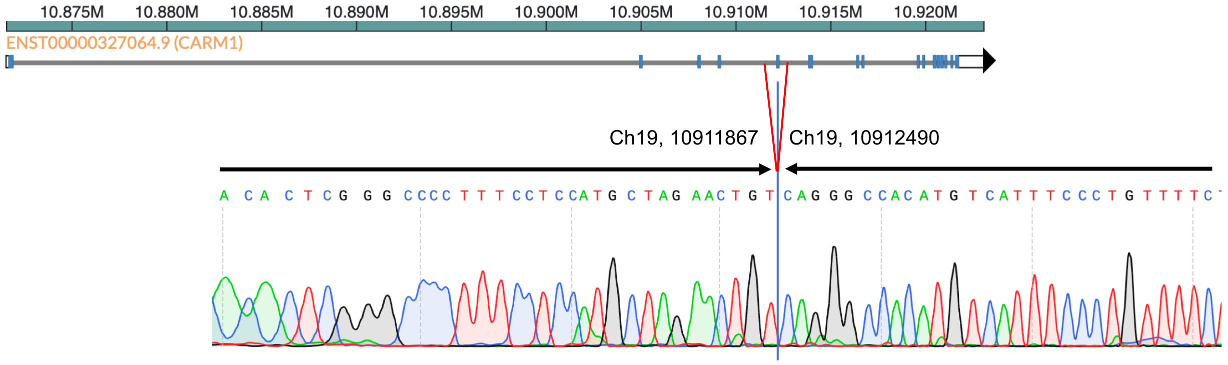

Genomic sequence of XPF KO HeLa cell

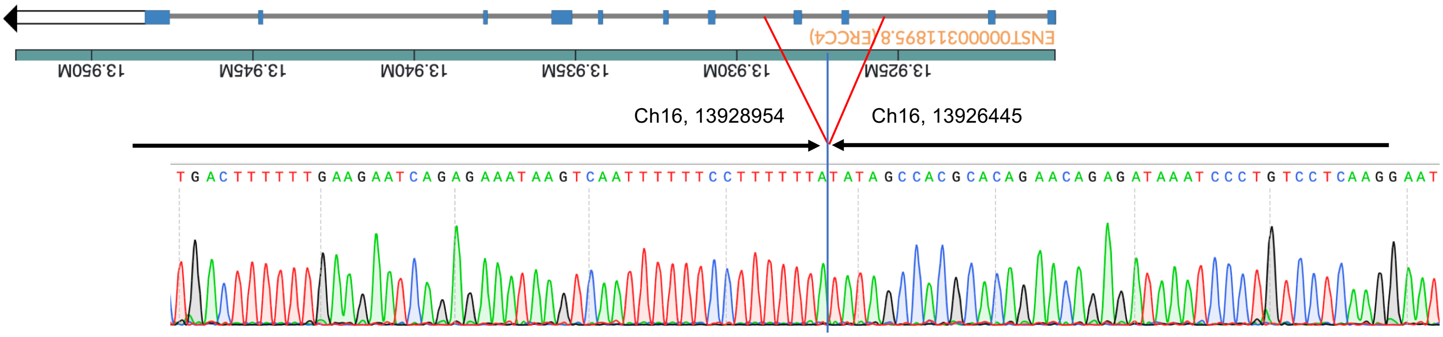

B

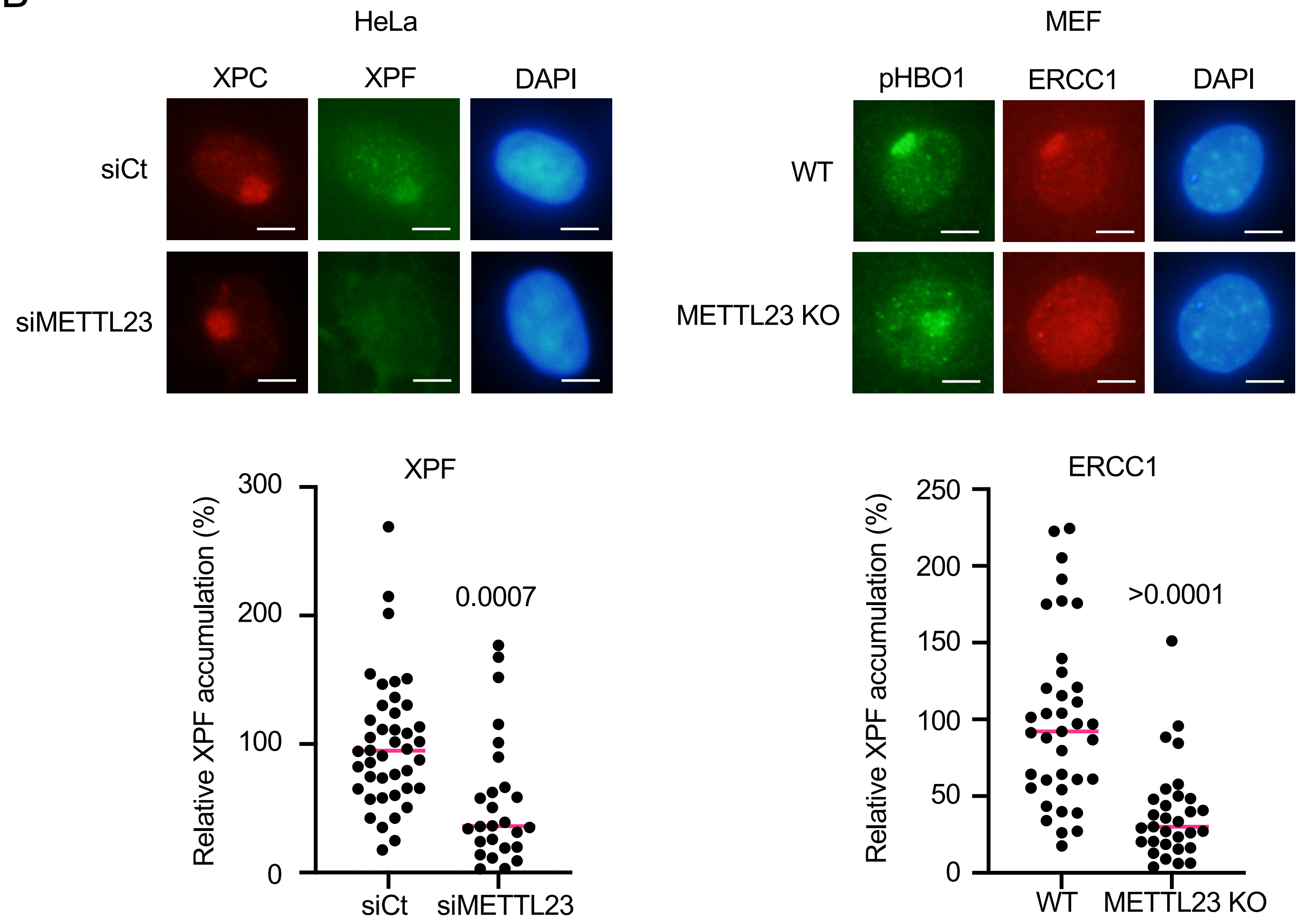

C

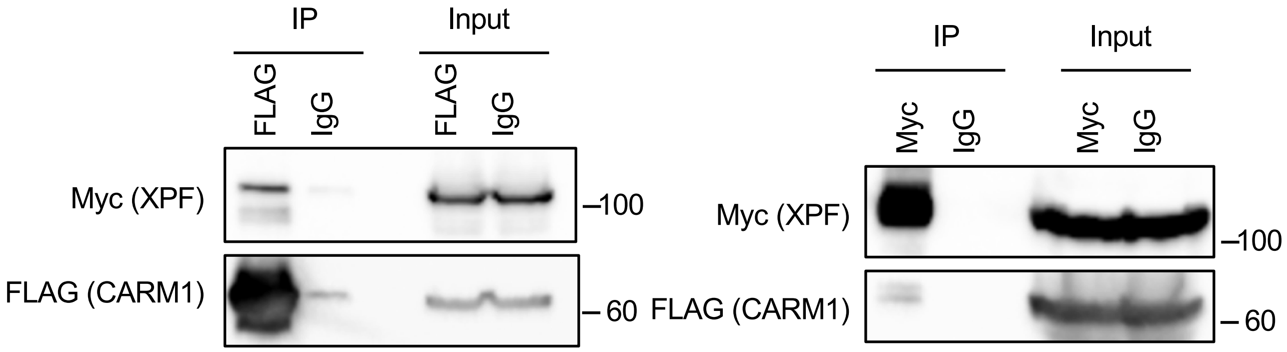
IP
Input
FLAG
IgG
FLAG
IgG
Myc (XPF)
FLAG (CARM1)
100
60
IP
Input
Myc
IgG
Myc
IgG
Myc (XPF)
FLAG (CARM1)
100
60

D

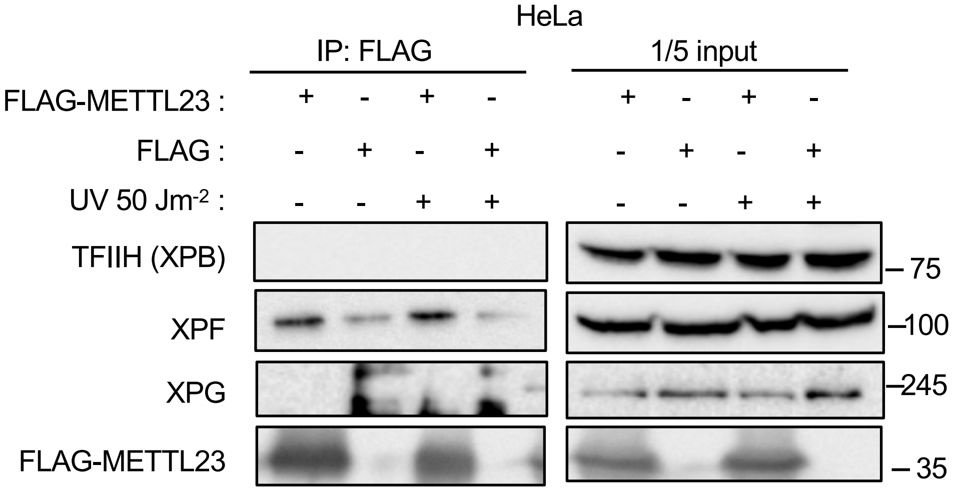
HeLa
IP: FLAG
1/5 input
FLAG-METTL23 :
FLAG :
UV 50 Jm-2 :
TFIIH (XPB)
XPF
XPG
FLAG-METTL23
75
100
245
35

Niida et al. Supplementary Fig. 3

A

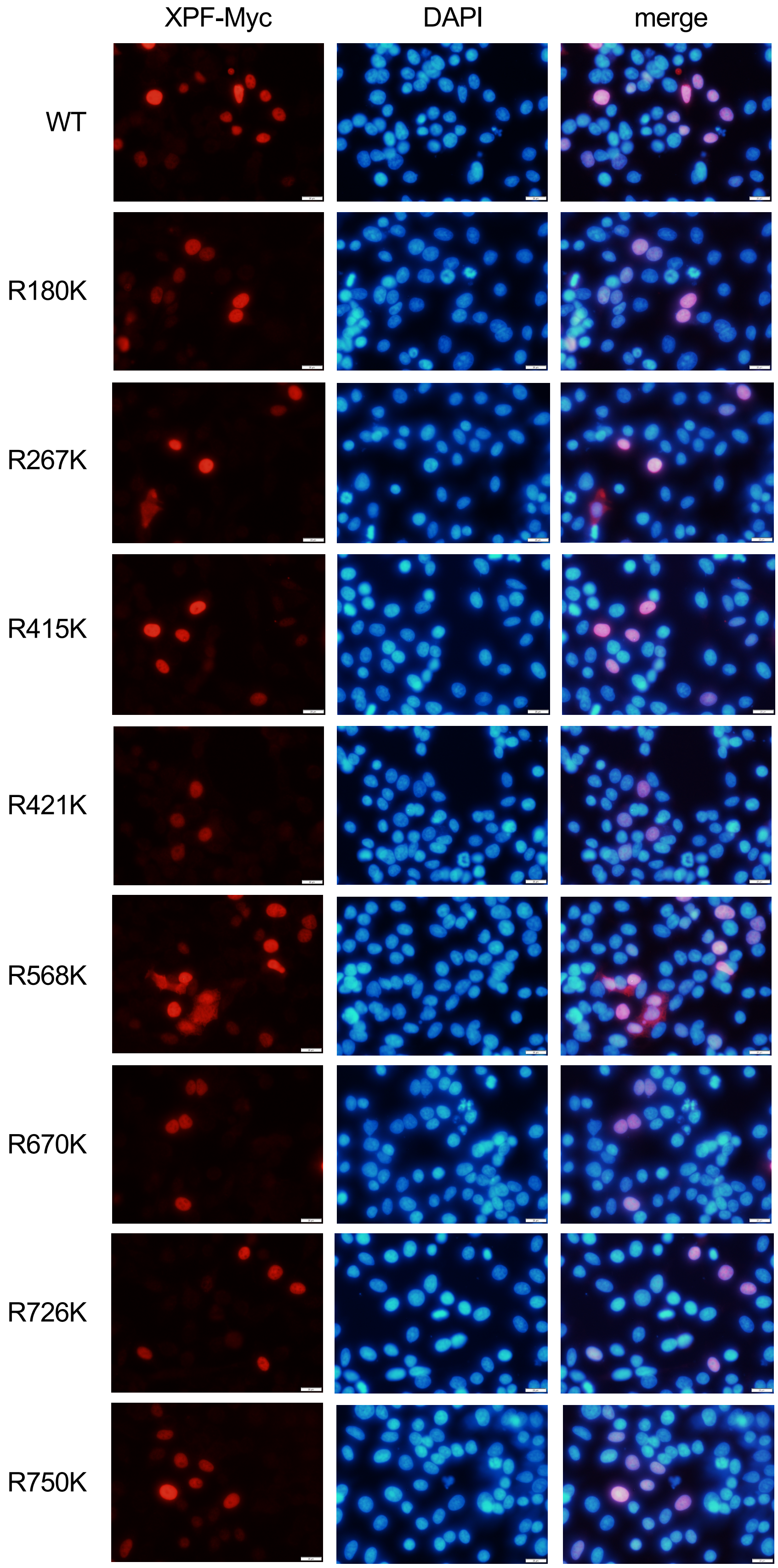

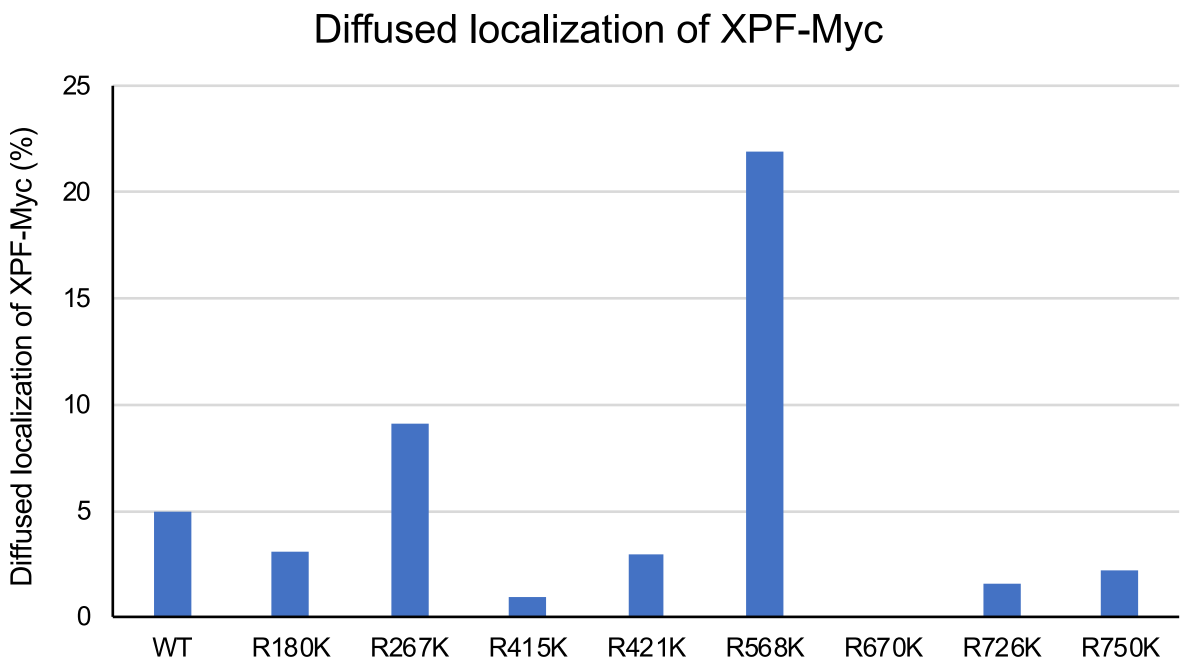

	nucleus	diffuse	%
WT	143	7	4.9
R180K	131	4	3.1
R267K	77	7	9.1
R415K	112	1	0.9
R421K	69	2	2.9
R568K	73	16	21.9
R670K	47	0	0.0
R726K	65	1	1.5
R750K	91	2	2.2

B

HeLa XPF KO

UV sensitivity

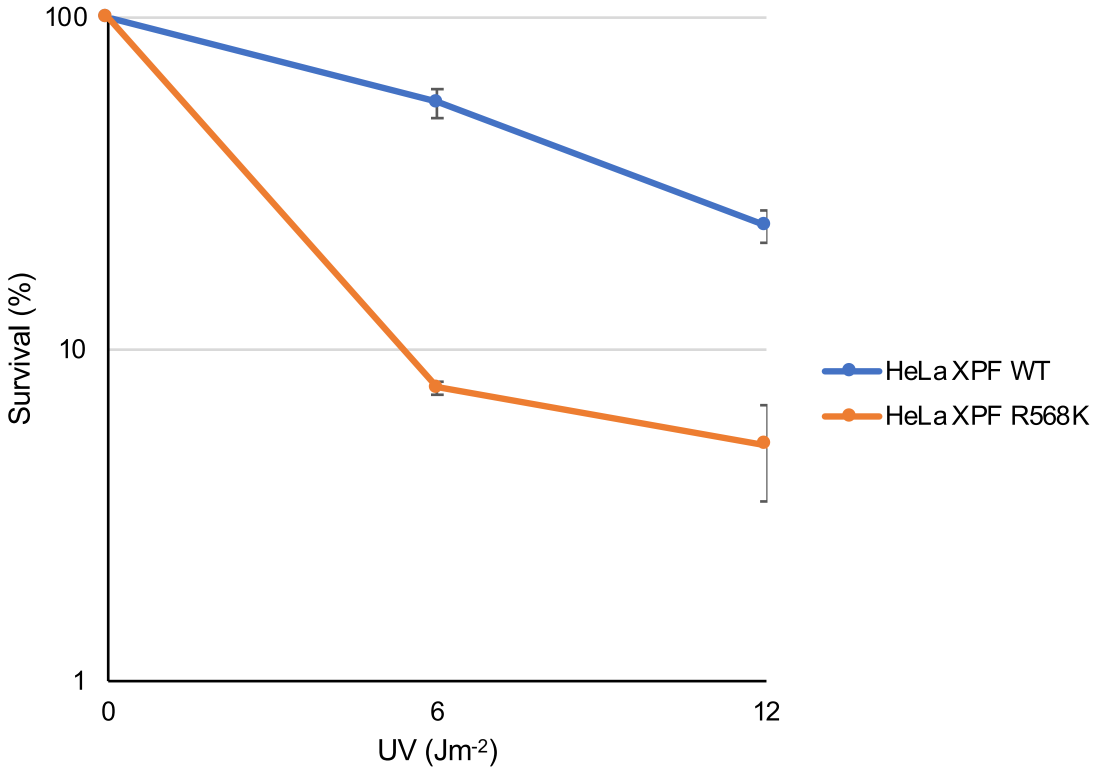

C

HeLa XPF KO

MMC sensitivity

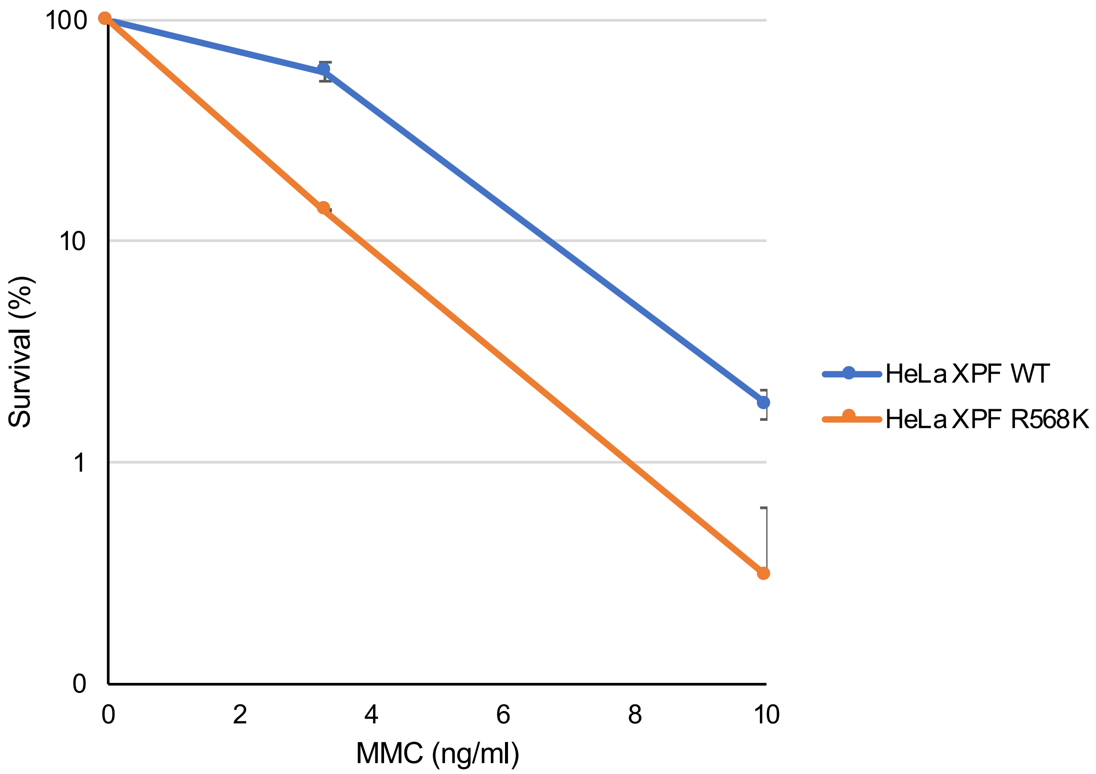

Niida et al. Supplementary Fig. 4

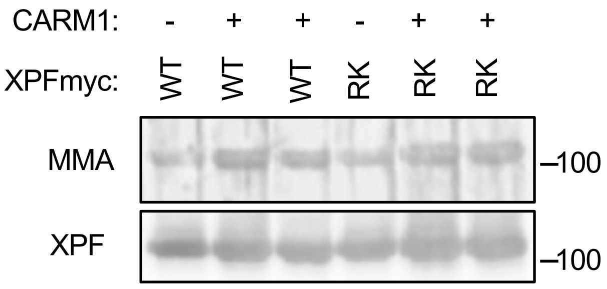

Supplement: gkaf355_Supplemental_Files [file gkaf355_supplemental_files.zip › Supplementary_figure_legends_2.pdf]
